# Maternal and neonatal outcomes of COVID-19 vaccination during pregnancy, a systematic review and meta-analysis

**DOI:** 10.1038/s41541-023-00698-8

**Published:** 2023-07-15

**Authors:** Greg Marchand, Ahmed Taher Masoud, Sandeep Grover, Alexa King, Giovanna Brazil, Hollie Ulibarri, Julia Parise, Amanda Arroyo, Catherine Coriell, Sydnee Goetz, Carmen Moir, Malini Govindan, Atley Moberly, Anna Proctor, Katelyn Sainz, Richard Blumrick

**Affiliations:** 1 Marchand Institute for Minimally Invasive Surgery, Mesa, AZ USA; 2 Faculty of Medicine, Fayoum University, Fayoum, Egypt; 3 Center for Human Genetics, Universitatsklinikum Giessen und Marburg, Marburg, Germany; 4 University of Iowa, College of Public Health, Iowa City, IA USA; 5 Tucson Medical Center, Department of Pediatrics, Tucson, AZ USA; 6 The Fetal Diagnostic Center, Mesa, AZ USA

**Keywords:** Outcomes research, Viral infection

## Abstract

Severe Acute Respiratory Syndrome Coronavirus 2 (SARS-CoV-2) is associated with increased pregnancy complications. Despite effective vaccination strategies for the general population, the evidence on the safety and efficacy of Coronavirus disease 2019 (COVID-19) vaccinations in pregnancy is limited due to a lack of well-powered studies. The present study compares the maternal, neonatal, and immunological outcomes between vaccinated pregnant and unvaccinated pregnant women using a systematic review and meta-analysis approach. We included 37 studies with a total of 141,107 pregnant women (36.8% vaccinated) spread across all outcomes. Our evidence indicates a higher rate of cesarean section in the 1898 vaccinated pregnant women compared to the 6180 women who did not receive vaccination (OR = 1.20, CI = (1.05, 1.38), *P* = 0.007, I2 = 45%). Regarding immunological outcomes, the risk of SARS-CoV-2 infection during pregnancy or postpartum was significantly reduced in 6820 vaccinated pregnant women compared to 17,010 unvaccinated pregnant women (OR = 0.25, CI = 0.13–0.48, *P* < 0.0001, *I*^2^ = 61%), as evident from qualitative assessment indicating significantly higher postpartum antibody titers compared to that observed in both unvaccinated mothers and mothers who have recently recovered from a SARS-CoV-2 infection. Our analysis represents high quality evidence showing that COVID-19 vaccination effectively raises antibody titers against SARS-CoV-2. This may confer protection against infection during pregnancy and the postpartum period. In addition to being protective against SARS-CoV-2, the vaccine was associated with decreased odds of preterm delivery. Furthermore, COVID-19 vaccination may also be associated with higher odds of cesarean section.

## Introduction

There is a growing body of evidence that vaccination against Severe Acute Respiratory Syndrome Coronavirus 2 (SARS-CoV-2) has the potential to considerably reduce the burden of Coronavirus disease 2019 (COVID-19)^1–3^. COVID-19 vaccines have also been shown to elicit strong protective responses in highly susceptible older adults and individuals with weak immune systems. A recent study has also found that pregnant women are more susceptible to presenting a more severe form of SARS-CoV-2 pneumonia than non-pregnant women and have higher rates of SARS-CoV-2-induced intensive care unit (ICU) admission, oxygen supplementations, need for mechanical ventilation, and death^3^. The virus can also affect neonatal outcomes, affecting up to 27% of infected mothers, who may suffer premature rupture of membranes, decreased fetal perfusion, and preterm births^4^. Other studies have shown that the incidence of preterm birth was tripled in symptomatic infected women compared to asymptomatic ones^5^. Also, blood hypercoagulability during SARS-CoV-2 infection increases the risk of thromboembolic events in pregnant women^6^. Studies have also reported an increased incidence of preeclampsia-like symptoms in infected mothers, such as hypertension, immune dysfunction, and thrombocytopenia without preeclampsia^7^. These complications and the severity of SARS-CoV-2 infection in pregnancy are likely due to the physiological changes in pregnant women, including increased cardiovascular requirements, decreased lung capacity, and immunological changes that are otherwise generally accepted to approximate a mildly immunocompromised state^8^.

Vaccination, in general, is usually safe during pregnancy, except for live attenuated vaccines^9,10^. However, the efficacy and safety of COVID-19 vaccination in pregnancy are unclear. Most COVID-19 vaccine trials failed to include pregnant women due to ethical concerns and the generalizability of the resulting data^11,12^. Animal data regarding the AstraZeneca vaccine’s usage in pregnancy has been reassuring, without any complications seen in comparable doses in mice^13^. In addition, data received from unintentionally vaccinated pregnant women from the COVID-19 immunization registry has thus far shown very few complications related to vaccine safety^14^. However, the lack of clinical trials still may make some women hesitant to receive COVID-19 vaccines, and this is an area where additional data is greatly needed^15^. Therefore, we performed this systematic review and meta-analysis to compare the maternal and neonatal outcomes between vaccinated pregnant and unvaccinated pregnant women, as well as to determine the antibody titers patterns of COVID-19 antibodies in both maternal and umbilical cord samples, and to determine the efficacy of vaccinations against COVID-19 administered during pregnancy to prevent COVID-19 infection.

## Results

### Study selection, study population characteristics, and quality assessment

**Fig. 1:**
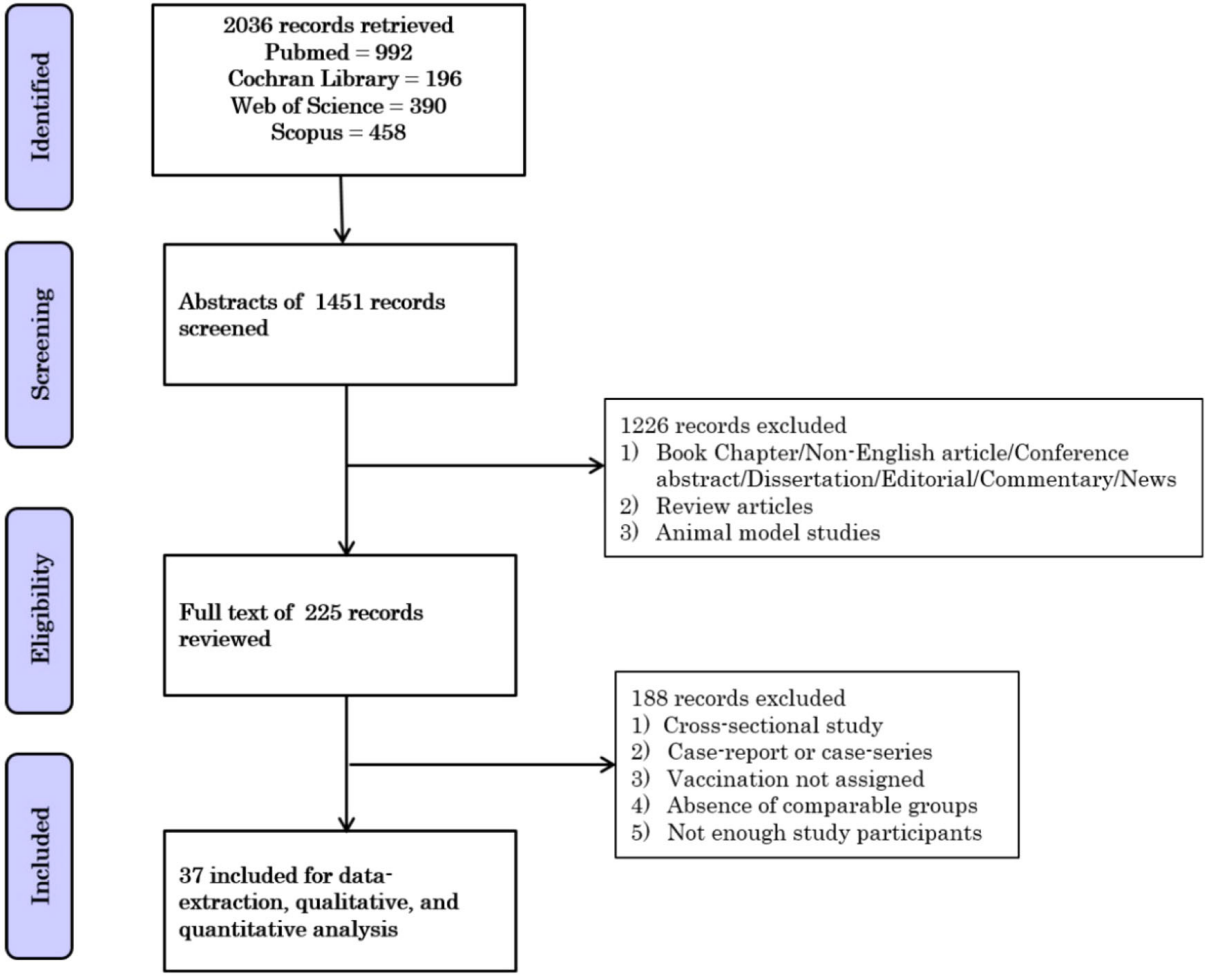
PRISMA workflow diagram.

Our database search resulted in 2036 records; 585 studies were removed as duplicates, and 1451 records remained for the title and abstract screening. Figure 1 shows the PRISMA flow diagram of our searching and screening processes. Two hundred twenty-five studies initially matched our inclusion criteria to enter the full-text screening process. Thirty-seven studies involving 141,107 pregnant women (36.8% vaccinated) were eligible for our systematic review and meta-analysis^14,16–51^. Characteristics of included studies are shown in Supplementary Table 1 and Supplementary Table 2. The majority of the included studies were cohort studies emanating from Israel and the United States. The most commonly administered vaccines were mRNA COVID-19 vaccines manufactured by Pfizer-BioNTech (BNT162b2) and Moderna (mRNA-1273). Among all the studies, only ten followed stringent criteria of defining vaccinated women as those who had received two doses of vaccination. All except a few studies reported the average age of the study participants between 30 and 35 years.

The overall mean result of the quality assessment was 8.75 (SD = 1.06), with none of the studies scoring <7 (low-bias) on a scale of 1-14. Notably, only one study reported data concerning blinding the outcome assessment^16^, and two provided sample size justification^24,28^. Supplementary Table 3 summarizes the results of the quality assessment.

### Meta-analysis

The results of the meta-analysis of the association of respective maternal, neonatal and immunological outcomes with vaccination status in pregnant women are summarized in Supplementary Table 4.

#### Maternal outcomes

**Fig. 2:**
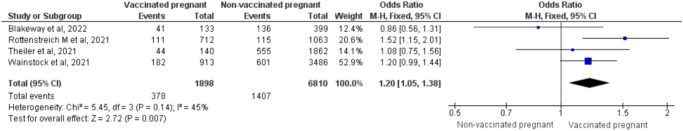
Forest Plot of the odds ratio of cesarean section in vaccinated pregnant women vs. unvaccinated pregnant women using Mantel-Haenszel.

Concerning maternal outcomes, we observed a significantly higher odds of cesarean section in 1898 vaccinated pregnant women compared to that observed in 6810 unvaccinated pregnant women s (OR = 1.20, 95% CI = 1.05–1.38, *P* = 0.007, *I*^2^ = 45%, *P*_het_ = 0.14; based on 4 studies)^18,43,46,48^ (Fig. 2).

None of the other outcomes, including maternal comorbidities, antepartum and postpartum complications, and duration of hospital stay at the time of delivery, showed association with vaccination administered during pregnancy (Supplementary Figs. S1–S8).

#### Neonatal outcomes

**Fig. 3:**
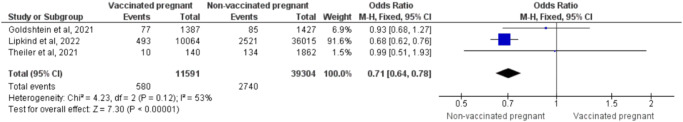
Forest Plot of the odds ratio of preterm birth in vaccinated pregnant women vs. unvaccinated pregnant women using Mantel-Haenszel.

Concerning neonatal outcomes, we observed a significantly lower odds of preterm birth in 11591 vaccinated pregnant women compared to that observed in 39304 unvaccinated pregnant women (OR = 0.71, 95% CI = 0.64–0.78, *P* < 0.00001, *I*^2^ = 53%, *P*_het_ = 0.12; based on three studies) (Fig. 3)^25,32,46^.

None of the other outcomes, including the incidence of a 5-min Apgar score ≤7, first-trimester miscarriage, fetal abnormalities, neonatal intensive care unit admission, small for gestational age, intrauterine growth restriction, and stillbirth, showed association with vaccination administered during pregnancy (Supplementary Figs. S9–S15).

#### Immunological outcomes

**Fig. 4:**
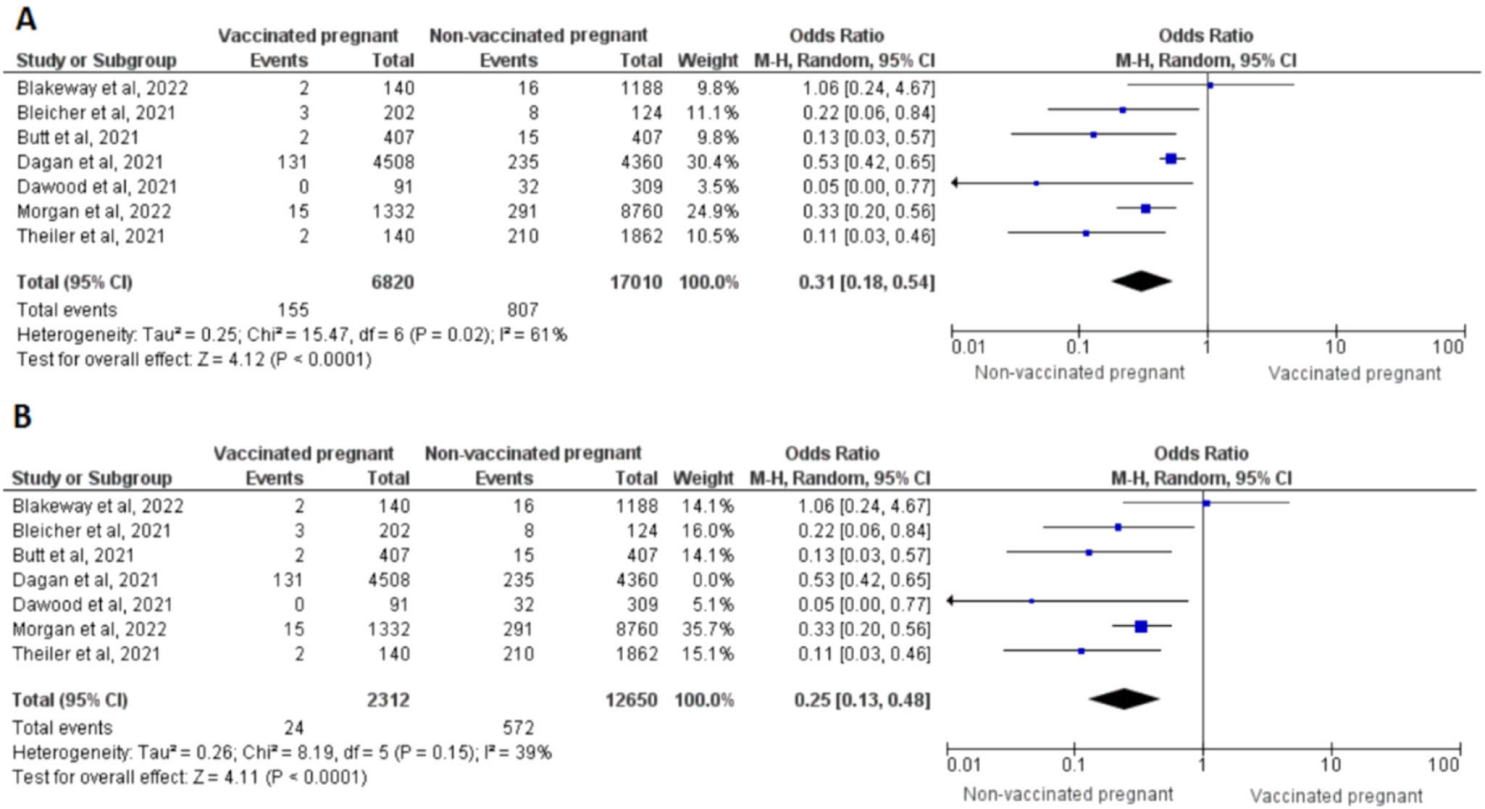
Forest plots are shown of the odds ratio of COVID-19 infection in vaccinated pregnant women vs. non-vaccinated pregnant women. Using Mantel-Haenszel, both before (**A**) and after (**B**) removing Dagan et al. to solve heterogeneity.

Concerning immunological outcomes, we could only pool the studies that investigated incidence of SARS-CoV-2 infection in vaccinated pregnant women. We observed significantly reduced incidence of SARS-CoV-2 infection in vaccinated pregnant women compared to that observed in unvaccinated pregnant women (OR = 0.31, 95% CI = 0.18–0.45, *P*_random_ < 0.0001, *I*^2^ = 61%, *P*_het_ = 0.02; based on 7 studies) (Fig. 4A)^18,20,23,24,28,36,46^. Our sensitivity analysis further identified study by Dagan et al.^23^. contributing to the heterogeneous results. However, exclusion of the study did not influence the overall significant association (OR = 0.25, 95% CI = 0.13–0.48, *P* < 0.0001, *I*^2^ = 39%, *P*_het_ = 0.15; based on 5 studies)^18,20,24,28,36,46^ (Fig. 4B).

### Qualitative synthesis

Due to the limited combinability of various immunological outcomes, including antibody levels and transfer ratios, we qualitatively synthesize the available evidence on the influence of vaccination on these outcomes.

## Immunological outcomes

### Maternal serum antibodies

#### Study characteristics

Sixteen studies assessed antibody titers in vaccinated maternal serum^16,17,19,22,26,30,31,35,38–42,44,49,51^. A complete summary of the study characteristics along with their findings are further shown in Supplementary Table 5. All studies reported an increase in IgG, IgM, and/or IgA in the sera of pregnant women after vaccination. The antibodies were assessed either to a receptor-binding domain (anti-RBD) and/or a spike protein. On the one hand, IgG was specifically strongly induced compared to IgM^35,39–42,44^. Gray et al.^26^. found that the highest increase in IgG levels occurred ~2–6 weeks after the second vaccination inoculation. On the other hand, while IgM and IgA were detected after the first dose, a significant increase in their levels was observed only after the second dose^26^. Notably, IgA levels were higher in women vaccinated with the mRNA-1273 vaccine than those vaccinated with the BNT162b2 vaccine^26^. Furthermore, anti-spike protein antibodies seemed to be induced more rapidly than anti-RBD antibodies^26^.

#### Primary findings

A few studies compared antibody levels in vaccinated pregnant women to unvaccinated pregnant women, showing a favorable response to vaccination^16,19,22,26,38,44,49^. For instance, Shanes et al. noted that IgG and IgM antibody levels were significantly higher in 52 vaccinated pregnant women than those observed in 116 unvaccinated pregnant women^44^. Several other studies reported higher antibody levels in vaccinated pregnant women than the unvaccinated pregnant women, who were known to have recovered from a recent SARS-CoV-2 infection^22,26,38^. Interestingly, stratification of IgG subtypes by Beharier et al. resulted in a more accurate assessment of immune response in vaccinated pregnant women. The authors observed higher levels of anti-RBD IgG and anti-spike S1 IgG and lower levels of anti-spike S2 IgG and anti-nucleocapsid IgG in vaccinated pregnant women^16^ simultaneously. These findings contrast with that reported by Yang et al., who failed to observe any difference in anti-spike IgG levels in vaccinated pregnant women compared to SARS-CoV-2 infected non-pregnant women, which could be due to a lack of information on the anti-spike subtype of IgG^49^. Some studies also investigated the influence of vaccination on antibody levels in pregnant women than that observed in non-pregnant women. We observed mixed findings; while some observed lower levels in pregnant women, others failed to detect any difference^19,22,26^.

#### Secondary findings

Several factors have been investigated that could play an essential role in determining efficacy of vaccination in pregnant women, including the number of vaccine doses administered and gestational age at the last vaccination. The administration of an additional or “booster” dose universally increases anti-spike IgG antibodies in fully vaccinated women^49^. Concerning the timing of vaccination, most studies found a positive correlation between levels of antibodies and gestational age of vaccination^41,49^. Yang et al. further observed a subsequent decline in anti-spike IgG after the 34th week of gestation^49^. By contrast, Gray et al. observed no significant correlation between the antibody levels and the trimester during which the vaccination was administered^26^. Maternal IgG levels at delivery were further dependent on the time passed since the first or second dose of the vaccine. For instance, Prahl et al. found no significant correlation between maternal IgG levels and time since the first dose administration, attributed to some participants receiving the first dose vaccine only 30 days prior to delivery^40^. Some studies also showed that the anti-RBD IgG at delivery correlated negatively with the time since the reception of the first^41^ or second vaccine^31,38^. Another study investigating pregnant women vaccinated with Johnson & Johnson vaccine did not show any difference in anti-spike protein IgG levels correlating with the timing of vaccination^49^.

### Fetal (Umbilical cord) blood antibody

#### Study characteristics

Sixteen studies assessed antibody titers in the umbilical cord blood of vaccinated mothers at the time of delivery^16,17,22,26,30,31,35,38–42,49,51^. Nearly all studies reported high levels of IgG in cord samples of vaccinated women irrespective of subtype (anti-RBD or anti-spike) assessed^17,22,26,30,31,35,38–42,49,51^. None of the studies detected IgM antibodies in the umbilical cord^17,35,41^. The IgG levels were lower in pregnant women who delivered after being administered the first dose of the vaccine than those who received both doses^26,35,39^. In a follow-up study, the dosage effect was further evident with IgG detection in only 9 of the 11 infants whose mothers received two doses prior to delivery (weeks of life range mean = 8.3). One of these infants continued to be positive for IgG at 12 weeks of age^40^.

#### Primary findings

Several reports have shown significantly higher levels of different antibody subtypes compared to unvaccinated pregnant women with no known history of SARS-CoV-2 infection^30^. Similar findings were observed when anti-RBD or anti-spike IgG levels in umbilical cord blood of neonates born to vaccinated women compared to those observed in unvaccinated SARS-CoV-2 infected pregnant women with a known recovery from the SARS-CoV-2 infection^22,30,38^. Another study failed to observe any difference in the distribution of anti-RBD or anti-S1 IgG between fully-vaccinated women compared to women with a prior SARS-CoV-2 infection^16^. Interestingly, the study also reported lower anti-nucleocapsid or anti-S1 IgG levels in fully vaccinated women^16^.

#### Secondary findings

Similar to the effect of additional dose administration on anti-spike IgG levels in sera of pregnant women with no history of SARS-CoV-2 infection, studies have reported significantly increased induction of anti-spike IgG in umbilical cord blood of vaccinated pregnant women compared to fully vaccinated women with negative history of SARS-CoV-2 infection. However, this difference is not retained when boosted vaccinated non-infected women were compared to women with a known history of SARS-CoV-2 infection and recovery^49^. Concerning the timing of vaccination, most studies found a positive correlation between levels of antibodies (anti-spike or anti-RBD IgG) and gestational age of vaccination^31,41,49^. The correlation was specifically stronger after the second dose of vaccination^31,41^. Yang et al. further observed that higher levels of antibodies were limited to the 32nd week of gestation, when their levels started declining^49^. The time since the first of the two administered doses of the vaccine also shows a positive correlation with anti-RBD and anti-spike IgG levels in umbilical cord blood^17,35,41,42,51^. However, the results showed considerable variation when the time since the second dose of the vaccine is considered. While Gray et al. observed a positive correlation between anti-spike IgG and time since administering the second dose of vaccine, Nir et al. failed to observe any correlation^26,38^. Another study reported a negative correlation between anti-RBD IgG levels and time since the administration of the second dose^31^.

### Transplacental antibody transfer ratio

Nine studies reported the transplacental antibody ratio (Cord/Maternal blood levels)^16,35,38–42,49,51^. Results varied widely between studies. While Zdanowski et al. reported a notably high transplacental antibody transfer ratio (mean ± SD = 1.28 ± 0.798)^51^, it was observed to be low for anti-RBD IgG by Nir et al. (median = 0.77)^38^ and Rottenstreich et al. (median = 0.34, IQR = 0.27–0.56)^42^. Another study reported a significantly lower transfer ratio for anti-S1 IgG, anti-S2 IgG, anti-RBD IgG in vaccinated pregnant women compared to previously infected women^16^. Generally, the transplacental ratio correlated positively with the time elapsed from vaccine dose to delivery^35,39–41,51^. Zdanowski et al. further observed a significantly higher transplacental ratio in early third-trimester vaccination than in late third-trimester vaccination^41^.

## Discussion

Overall, our systematic review and meta-analysis show that the administration of the COVID-19 vaccine to pregnant women is safe and effective. The odds of SARS-CoV-2 infection are significantly lower in the vaccinated group, compared to unvaccinated women. This evidence supports guidelines from major groups recommending universal vaccination during pregnancy. Our finding of decreased odds of preterm delivery in vaccinated women is not surprising as several studies have demonstrated the connection between SARS-CoV-2 infection in pregnancy and preterm delivery^52,53^. It is notable however, when taking into account the small number of COVID-19 cases in the unvaccinated population, that some of the tendency to deliver early may be iatrogenic, especially earlier in the pandemic. The odds of cesarean delivery are also significantly higher in the vaccinated group, while no clear explanation exists for this phenomenon. Other than these results, we found no significant differences between the vaccinated and unvaccinated groups concerning maternal and neonatal outcomes. Unfortunately, secondary to varying definitions of “fully vaccinated” in the different included studies, we could not carry out stratified analysis for each of these definitions due to lack of power to perform such an analysis. It should be noted that we adopted the random effects model to help account for these heterogeneous findings.

Concerning maternal humoral immunity, although factors related to combinability limited the possibility of a quantitative synthesis, we can report several findings. First, all pregnant women who received any vaccination showed high anti-RBD or anti-spike protein antibody levels. There was no evidence of ineffective vaccination. In all cases, the level of IgG was higher than IgM^39,40,54,55^. In all cases, vaccinated pregnant women showed higher titers of anti-RBD antibodies when compared to previously infected unvaccinated women^56^. On the other hand, unvaccinated SARS-CoV-2 infected pregnant women showed lower antibodies titers when compared to vaccinated pregnant, lactating, and non-pregnant women^22^. Meanwhile, the vaccinated women had significantly higher anti-RBD IgG and anti-S1 IgG levels than infected women, while significantly higher levels of an anti-S2 segment of spike protein IgG and anti-Nucleocapsid IgG antibodies in infected women compared to vaccinated women^16^. The antibody response was affected by additional or “booster” dose administration, the trimester time of vaccine administration, and the time passed from the first or second vaccine dose^39,49,55^. Administration of the second vaccine dose to the women achieved higher titers of anti-RBD and anti-spike protein IgG in umbilical cord samples of the neonates compared to those born to women who took the first dose only^54^. Non-infected unvaccinated women had negative antibody samples, while vaccinated pregnant women showed high antibody titers^57^. Neonates born to vaccinated women showed higher anti-RBD and anti-spike protein IgG titers than those born to SARS-CoV-2 infected unvaccinated women^22^. As for transplacental antibody transfer, SARS-CoV-2 infected women had a higher IgG transfer ratio for the anti-S1 segment of spike protein, the anti-S2 segment of spike protein, and anti-RBD than the vaccinated, negative anti-Nucleocapsid group; however, no significant difference was found between positive anti-Nucleocapsid vaccinated women and the other two groups^16^.

Several studies have highlighted that vaccinated or infected pregnant women can transfer antibodies against SARS-CoV-2 to the fetus. The vaccination specifically generates anti-spike IgG antibodies which have been detected in the umbilical cord, Furthermore, the antibodies continue to be detected in infants after birth. A recent study of 28 infants of vaccinated mothers reported significantly higher titers at 6 months in 16 infants (57%)^58^. On the other hand, only 1 of 12 infants (8%) born to infected mothers had detectable antibodies at 6 months. However, IgM antibodies have not been detected in cord blood samples indicating that they do not cross the placenta, which could be attributed to their large macromolecular structure^59^. Antibodies in the milk from lactating women who had received COVID-19 vaccine have been shown to neutralize spike and several variants of concern^60^. The immune response to maternal vaccination was also reflected in detection of antibodies in 1/3rd of breastfed infant stool^60^.

Similar to our study results, Pratma et al., in their meta-analysis and systematic review, found that the administration of mRNA vaccine to pregnant women effectively reduced the incidence of further SARS-CoV-2 infections and provided antibody response to the pregnant women and their fetuses which was increased by administration of a second vaccine dose. Additionally, they found the vaccine-induced higher antibody titers compared to that produced by SARS-CoV-2 infection without vaccination. They also found that the vaccine had no significant effect on maternal, delivery, and neonatal outcomes^61^. On the contrary, we found that the vaccinated group had significantly lower odds of preterm delivery and higher odds of cesarean section than the non-infected unvaccinated group. Other reviews and meta-analyses demonstrated the efficacy and safety of the COVID-19 vaccine for pregnant women and found that the administration of the vaccine to pregnant women was safe and effective. Many also recommended administering an additional or “booster” dose, which may induce a higher antibody response^62–64^. These results agreed with our study results, but our analysis included the largest number of studies. Ma et al. included only six studies^64^, Fu et al. included 23 studies^63^, Pratama et al. included 12 studies^62^, while our study included 37 studies. Moreover, our analysis illustrated the factors affecting antibody response for mothers and their fetuses in more detail.

Also in agreement with our findings are some of the results of the recently published Dick et al.^65^. This 2022 retrospective cohort study compared pregnant women of different vaccination statuses during pregnancy. They found, similar to our results, a slightly higher rate of gestational diabetes among those women vaccinated against COVID-19. There was also a slightly increased rate of delivery of Cesarean deliveries amongst the triple vaccinated compared to the non-vaccinated, also similar to the present study. It will be interesting to see if these small differences exist in future studies and what causes they could be attributed to.

There are some obstacles to managing the SARS-CoV-2 pandemic and achieving herd immunity. Acceptance of COVID-19 vaccination is an important one of these issues. Wake et al. found that acceptance of the vaccine was very low in Africa^66^. Tomasz et al. found that the vaccine’s acceptance rate among pregnant women ranges between 29.7% and 77.4%. This range depends on many factors, the most important of which are the awareness of the infection, the safety of the vaccine, and the way to provide information about the vaccine and its safety^67^. It should be noted, however, that because of the absence of follow-up data for both mother and the infant, long term safety and efficacy of COVID-19 vaccines cannot be judged using only the present study. Developing resistance among SARS-COV-2 variants represents another problem that should be taken into consideration^68,69^. Moreover, there are mutations in some variants that affect the virus’s transmissibility and severity. These mutations also affect the COVID-19 treatment efficacy^62,70^. This problem could create a need for additional vaccine formulations to better protect populations^71^. We consider the COVID-19 pandemic to be a rapidly evolving situation with a constant need of updating vaccines to target newer variants of concern. Although the findings from the present study may not be directly applicable to the current pandemic, they will likely help in designing better vaccines in future.

The main strength of our comprehensive meta-analyses is the incorporation of the latest studies resulting in the largest sample size reported to date for multiple study outcomes. However, the inclusion of observational studies which were often unmatched continues to be the main limitation in judging the evidence, especially in light of the fact that the included observational trials largely gave only immunological, not clinical outcomes. Furthermore, considerable heterogeneity in vaccine type, dosing and schedule, and measurement of different antibody subtypes, often made the comparison and pooling of various outcomes highly challenging. In addition, there is always the possibility that IgG levels may be under-estimated in the serum of the study participation. If future studies were to measure the SARS-CoV-2 antibody in the saliva instead, this may provide a better measurement of the degree of protective immunity against COVID-19. Another limitation included the fact that our study fails to account for the role of T-cells and innate immunity. It is possible that a large majority of uninfected adults may already have pre-existing antibodies against SARS-CoV-2, which would decrease the accuracy of our findings. In addition, because of the lack of details surrounding the timing of vaccination, our present study cannot account for the waning of immunity over the course of a pregnancy. One final limitation has to do with the effect that high levels of stress may have on a pregnant mother during a pandemic, particularly on mothers who have made the decision to defer vaccination against the advice of their physician. As authors we see no effective way to control for the effect of this stress on our outcomes. Nevertheless, our study provides various novel insights into potential influence of vaccination on various novel outcomes. Our study further emphasizes a need to increase the awareness about the SARS-CoV-2 infection and the safety of vaccine administration to pregnant women, through obstetricians and medical personnel.

## Methods

We performed our systematic review and meta-analysis according to the Preferred Reporting Items for Systematic Reviews and Meta-analysis (PRISMA) guidelines, Meta-analysis Of Observational Studies in Epidemiology (MOOSE), and the Cochrane handbook of systematic review and meta-analysis of interventions^72–75^.

### Systematic literature search and screening of articles

We searched six databases: PubMed, Scopus, Medline, Cochrane Library, and Web of Science for relevant observational studies conducted on COVID-19 vaccinated pregnant women by using a combination of Medical Subject Headings (MeSH) terms and keywords to formulate this strategy: ((COVID vaccine OR COVID19 vaccine OR COVID immunization OR COVID-19 Virus Vaccine OR SARS CoV 2 Vaccines OR SARS-CoV-2 Vaccine OR SARS CoV 2 Vaccine OR SARS2 Vaccine OR Coronavirus Disease 2019 Vaccine OR 2019 nCoV Vaccine OR 2019 Novel Coronavirus Vaccines OR HIPRA SARS-CoV-2 vaccine OR Gam-COVID-Vac vaccine OR Ad5-nCoV vaccine OR HDT-301 vaccine OR MVC-COV1901 vaccine OR recombinant SARS-CoV-2 vaccine NVX-cov2373 OR Covid-19 aAPC vaccine OR lentiviral minigene vaccine OR COVAC-1 vaccine OR COVID-19 Vaccines OR 2019-nCoV Vaccine mRNA-1273 OR BNT162 Vaccine OR ChAdOx1 nCoV-19 OR Ad26COVS1 OR EpiVacCorona vaccine OR ChulaCov19 vaccine) AND (Pregnancy OR pregnant OR gestation OR gravidity OR childbearing)). We searched all English language papers from each database’s inception until January 31st, 2022, followed by manual searching of citations of the shortlisted articles to identify any additional articles. The screening process was conducted in two phases according to the eligibility criteria and was performed in parallel by two investigators (GM and ATM). The first phase comprised screening of title and abstract, followed by the next phase of full-text screening based on inclusion and exclusion criteria, as mentioned in the next section. Any conflict about the eligibility of a specific study was resolved by involving a third investigator (SG).

### Study selection

We included all studies conducted on pregnant women receiving COVID-19 vaccination of any mechanism of action approved by an accredited body. The eligibility criteria for the included studies were: (I) Population: vaccinated pregnant women. (II) Intervention: any COVID-19 Vaccine. (III) Comparator: unvaccinated pregnant women (IV) Outcomes: (a) Maternal: unassisted vaginal delivery, operative vaginal delivery, cesarean section, gestational diabetes, gestational hypertension, preeclampsia, placental abruption, postpartum hemorrhage (defined as >500cc blood loss), length of hospital stay at the time of delivery; (b) Neonatal: preterm delivery, 5-min Apgar score ≤7, first-trimester miscarriage, fetal abnormalities, neonatal intensive care unit admission, small for gestational age (defined as less than the 10th percentile weight at birth), and intrauterine growth restriction (defined as fetal weight estimated to be less than the 10th percentile for weight at any point prior to delivery), (c) Immunological: maternal COVID-19 infection confirmed by COVID-19 by a polymerase chain reaction (PCR) test or with ICD-10 codes for confirmed COVID-19 at any time in the antepartum or postpartum period (defined as the entire pregnancy to 6 weeks postpartum), and vaccination antibody levels at delivery of both mother and infant, and (V) Study design: We included all observational studies including cohort and case-control studies. We excluded reviews, case reports, case series, studies with lack of comparable data, and studies that reported data on less than ten vaccinated pregnant women. We further used only the latest dataset published by a group, to avoid duplication of samples.

### Data extraction and quality assessment

We extracted the information on study design, demographic and baseline characteristics of the study populations, including maternal age, gestational age, BMI, gravidity, parity, the incidence of pre-gestational diabetes, and pre-gestational hypertension. In addition, we extracted the data for our selected outcomes for statistical analysis (See outcomes in the section on Inclusion and Exclusion criteria). We used the National Heart, Lung, and Blood Institute (NHLB) tool for the quality assessment tool of observational studies, including 14 domains assessing the overall quality of the studies^76^.

### Meta-analysis and qualitative synthesis

We analyzed the extracted outcomes using Review Manager Software (Version 5.0). Dichotomous outcomes were meta-analyzed using Odds Ratio (OR) and continuous data using Mean Difference (MD). We used forest plots to show individual and pooled effect estimates with 95% confidence intervals (CI). We performed the meta-analysis comparing vaccinated to unvaccinated pregnant women with no history of SARS-CoV-2 infection by using the Mantel-Haenszel for categorical outcomes and Inverse Variance for continuous outcomes, the results were considered significant if the *P*-value for overall effect was <0.05. In general, we employed a fixed-effects model as a default to pool the individual effect estimates (OR or MD). A random-effects model was only used for heterogeneous findings (if *P*_het_ < 0.1 as well as I2 > 50%)^77^. We conducted sensitivity analysis by the “leave-one-out” method to identify sources of heterogeneity and reliability of our findings^77^.

Due to limited combinability in specific immunological outcomes, such as different titer measurement protocols, we qualitatively summarized the outcomes related to antibody levels as maternal serum antibody levels, umbilical cord sample antibody levels, and transfer ratios. A quantitative synthesis was not possible for those results. Also, we qualitatively compared these levels in vaccinated pregnant women to those in infected unvaccinated pregnant women, non-pregnant vaccinated women, and non-infected unvaccinated women. Finally, we studied the specific factors that influenced each outcome, including additional or “booster” dose administration, gestational vaccination time, and time elapsed from the first or second dose until delivery.

## Conclusion

In conclusion, we found that the administration of the COVID-19 vaccine to pregnant women effectively prevented future SARS-CoV-2 infection. It appears safe and seems to provide passive immunity to neonates. Administration of an additional or “booster” dose may increase the immune response and transplacental antibody transfer. As this data applies to an actively evolving pandemic, changes in the clinical scenario, including variations of the virus and changes in the immune status of affected populations will invariably limit how applicable this data is to the day-to-today prevention and treatment of COVID-19. In addition to being protective against SARS-CoV-2, the vaccine was associated with decreased odds of preterm delivery. Also, for reasons not entirely understood, our data shows that COVID-19 vaccination may also be associated with higher odds of cesarean section.

## Supplementary information

Supplemental Material

## Data Availability

As the work presented is a meta analysis, all data used is available from the original authors of the compiled studies online. Prospero Registration: CRD42022324416.

## References

[1] Polack FP (2020). Safety and efficacy of the BNT162b2 mRNA COVID19 vaccine. N. Engl. J. Med..

[2] Baden LR (2021). Efficacy and safety of the mRNA-1273 SARS-CoV-2 Vaccine. N. Engl. J. Med..

[3] Zambrano LD (2020). Update: Characteristics of symptomatic women of reproductive age with laboratory-confirmed SARS-CoV-2 infection by pregnancy status—United States, January 22–October 3, 2020. MMWR Morb. Mortal. Wkly Rep..

[4] Dubey P (2020). Maternal and neonatal characteristics and outcomes among COVID19 infected women: an updated systematic review and meta-analysis. Eur. J. Obstet. Gynecol. Reprod. Biol..

[5] Delahoy MJ (2020). Characteristics and maternal and birth outcomes of hospitalized pregnant women with laboratory-confirmed COVID19—COVID-NET, 13 States, March 1–August 22, 2020. MMWR Morb. Mortal. Wkly Rep..

[6] Benhamou D, Keita H, Ducloy-Bouthors AS (2020). Coagulation changes and thromboembolic risk in COVID19 obstetric patients. Anaesth., Crit. Care Pain. Med.

[7] Mendoza M (2020). Pre-eclampsia-like syndrome induced by severe COVID19: a prospective observational study. BJOG.

[8] Mertz D (2013). Populations at risk for severe or complicated influenza illness: systematic review and meta-analysis. BMJ.

[9] Munoz FM (2014). Safety and immunogenicity of Tetanus diphtheria and acellular pertussis (Tdap) immunization during pregnancy in mothers and infants: a randomized clinical trial. JAMA.

[10] Lu QC (2021). One ‘misunderstood’ health issue: demonstrating and communicating the safety of influenza A vaccination in pregnancy: a systematic review and meta-analysis. BMC Public Health.

[11] Randolph HE, Barreiro LB (2020). Herd immunity: understanding COVID19. Immunity.

[12] Rasmussen SA (2021). Coronavirus disease 2019 (COVID19) vaccines and pregnancy: what obstetricians need to know. Obstet. Gynecol..

[13] Stebbings R (2021). Developmental and reproductive safety of AZD1222 (ChAdOx1 nCoV-19) in mice. Reprod. Toxicol..

[14] Shimabukuro TT (2021). Preliminary findings of mRNA COVID19 vaccine safety in pregnant persons. N. Engl. J. Med..

[15] Skjefte M (2021). COVID19 vaccine acceptance among pregnant women and mothers of young children: results of a survey in 16 countries. Eur. J. Epidemiol..

[16] Beharier, O. et al. Efficient maternal to neonatal transfer of antibodies against SARS-CoV-2 and BNT162b2 mRNA COVID19 vaccine. *J. Clin. Invest*. 10.1172/JCI150319 (2021).

[17] Ben-Mayor Bashi T (2022). The association of maternal SARS-CoV-2 vaccination-to-delivery interval and the levels of maternal and cord blood antibodies. Int J. Gynecol. Obstet..

[18] Blakeway H (2022). COVID19 vaccination during pregnancy: coverage and safety. Am. J. Obstet. Gynecol..

[19] Bookstein Peretz S (2021). Short-term outcome of pregnant women vaccinated with BNT162b2 mRNA COVID19 vaccine. Ultrasound Obstet. Gynecol..

[20] Butt, A. A. et al. SARS-CoV-2 vaccine effectiveness in preventing confirmed infection in pregnant women. *J. Clin. Invest*. **131**. 10.1172/JCI153662 2021. 10.1172/JCI153662 PMC8631593 34618693

[21] Atyeo, C. et al. COVID19 mRNA vaccines drive differential antibody Fc-functional profiles in pregnant, lactating, and nonpregnant women. *Sci. Transl. Med*. 10.1126/scitranslmed.ABI8631 (2021). 10.1126/scitranslmed.abi8631 PMC9067624 34664972

[22] Collier ARY (2021). Immunogenicity of COVID19 mRNA vaccines in pregnant and lactating women. JAMA - J. Am. Med Assoc..

[23] Dagan N (2021). Effectiveness of the BNT162b2 mRNA COVID19 Vaccine in Pregnancy. Nat. Med.

[24] Dawood F. S. et al. Incidence, clinical characteristics, and risk factors of Sars-cov-2 infection among pregnant individuals in the United States. *Clin. Infect. Dis*. **74**, 2218–2226 (2021). 10.1093/cid/ciab713 PMC8513407 34410340

[25] Goldshtein I (2021). Association between BNT162b2 vaccination and incidence of SARS-CoV-2 infection in pregnant women. JAMA.

[26] Gray KJ (2021). Coronavirus disease 2019 vaccine response in pregnant and lactating women: a cohort study. Am. J. Obstet. Gynecol..

[27] Hillson K (2021). Fertility rates and birth outcomes after ChAdOx1 nCoV-19 (AZD1222) vaccination. Lancet.

[28] Bleicher I, Kadour-Peero E, Sagi-Dain L, Sagi S (2021). Early exploration of COVID-19 vaccination safety and effectiveness during pregnancy: interim descriptive data from a prospective observational study. Vaccine.

[29] Kachikis A (2021). Short-term reactions among pregnant and lactating individuals in the first wave of the COVID19 vaccine rollout. JAMA Netw. Open.

[30] Kashani-Ligumsky L (2021). Titers of SARS CoV-2 antibodies in cord blood of neonates whose mothers contracted SARS CoV-2 (COVID19) during pregnancy and in those whose mothers were vaccinated with mRNA to SARS CoV-2 during pregnancy. J. Perinatol..

[31] Kugelman, N. et al. Maternal and neonatal SARS-CoV-2 immunoglobulin G antibody levels at delivery after receipt of the BNT162b2 messenger RNA COVID19 vaccine during the second trimester of pregnancy. *JAMA Pediatr*. 10.1001/jamapediatrics.2021.5683 (2021). 10.1001/jamapediatrics.2021.5683 PMC8693209 34932066

[32] Lipkind HS (2022). Receipt of COVID19 vaccine during pregnancy and preterm or small-for-gestational-age at birth—eight integrated health care organizations, United States, December 15th, 2020–July 22nd, 2021. MMWR Morb. Mortal. Wkly Rep..

[33] Magnus MC (2021). COVID19 vaccination during pregnancy and first-trimester miscarriage. N. Engl. J. Med.

[34] Matsui, Y. et al. Neutralizing antibody activity against SARS-CoV-2 variants in gestational age–matched mother-infant dyads after infection or vaccination. *JCI Insight*. **7**, e157354 (2022). 10.1172/jci.insight.157354 PMC9309042 35579965

[35] Mithal LB (2021). Cord blood antibodies following maternal coronavirus disease 2019 vaccination during pregnancy. Am. J. Obstet. Gynecol..

[36] Morgan JA (2022). Maternal outcomes after severe acute respiratory syndrome coronavirus 2 (SARS-CoV-2) infection in vaccinated compared with non-vaccinated pregnant patients. Obstet. Gynecol..

[37] Nakahara, A. et al. Safety-related outcomes of novel mRNA COVID19 vaccines in pregnancy. *Am. J. Perinatol*. 10.1055/a-1745-1168 (2022). 10.1055/a-1745-1168 35045574

[38] Nir O (2022). Maternal-neonatal transfer of SARS-CoV-2 immunoglobulin G antibodies among parturient women treated with BNT162b2 messenger RNA vaccine during pregnancy. Am. J. Obstet. Gynecol. MFM.

[39] Prabhu M (2021). Antibody response to coronavirus disease 2019 (COVID19) messenger RNA vaccination in pregnant women and transplacental passage into cord blood. Obstet. Gynecol..

[40] Prahl M (2022). Evaluation of transplacental transfer of mRNA vaccine products and functional antibodies during pregnancy and infancy. Nat. Commun..

[41] Rottenstreich, A. et al. Timing of SARS-CoV-2 vaccination during the third trimester of pregnancy and transplacental antibody transfer: a prospective cohort study. *Clin. Microbiol. Infect*. 10.1016/j.cmi.2021.10.003 (2021). 10.1016/j.cmi.2021.10.003 PMC8563509 34740773

[42] Rottenstreich A (2021). Efficient maternofetal transplacental transfer of anti- severe acute respiratory syndrome coronavirus 2 (SARS-CoV-2) spike antibodies after antenatal SARS-CoV-2 BNT162b2 messenger RNA vaccination. Clin. Infect. Dis..

[43] Rottenstreich M (2022). COVID19 vaccination during the third trimester of pregnancy: rate of vaccination and maternal and neonatal outcomes, a multicentre retrospective cohort study. BJOG Int. J. Obstet. Gynaecol..

[44] Shanes ED (2021). Severe acute respiratory syndrome coronavirus 2 (SARS-CoV-2) vaccination in pregnancy: measures of immunity and placental histopathology. Obstet. Gynecol..

[45] Shen CJ (2022). Evaluation of transplacental antibody transfer in SARS-CoV-2-immunized pregnant women. Vaccines.

[46] Theiler RN (2021). Pregnancy and birth outcomes after SARS-CoV-2 vaccination in pregnancy. Am. J. Obstet. Gynecol. MFM.

[47] Trostle ME (2021). COVID19 vaccination in pregnancy: early experience from a single institution. Am. J. Obstet. Gynecol. MFM.

[48] Wainstock T (2021). Prenatal maternal COVID19 vaccination and pregnancy outcomes. Vaccine.

[49] Yang YJ (2022). Association of gestational age at coronavirus disease 2019 (COVID19) vaccination, history of severe acute respiratory syndrome coronavirus 2 (SARS-CoV-2) infection, and a vaccine booster dose with maternal and umbilical cord antibody levels at delivery. Obstet. Gynecol..

[50] Zauche, L. H. et al. Receipt of mRNA COVID19 vaccines preconception and during pregnancy and risk of self-reported spontaneous abortions, CDC V-safe COVID19 vaccine pregnancy registry 2020–21. *Res. Sq.*10.21203/rs.3.rs-798175/v1 (2021).

[51] Zdanowski W, Waśniewski T (2021). Evaluation of Sars-cov-2 spike protein antibody titers in cord blood after COVID19 vaccination during pregnancy in polish healthcare workers: preliminary results. Vaccines.

[52] Martinez-Perez O (2021). The association between SARS-CoV-2 infection and preterm delivery: a prospective study with a multivariable analysis. BMC Pregnancy Childbirth.

[53] Bobei TI (2022). The impact of SARS-CoV-2 infection on premature birth—our experience as COVID center. Medicina.

[54] Shanes, E. D. et al. Severe acute respiratory syndrome in pregnancy measures of immunity and placental histopathology. *Obstet. Gynecol*. 10.1111/aji.13332.2 2021. 10.1097/AOG.0000000000004457 PMC8288194 33975329

[55] Rottenstreich A (2021). Efficient maternofetal transplacental transfer of anti- severe acute respiratory syndrome coronavirus 2 (SARS-CoV-2) spike antibodies after antenatal SARS-CoV-2 BNT162b2 messenger RNA vaccination. Clin. Infect. Dis..

[56] Gray KJ (2021). COVID-19 vaccine response in pregnant and lactating women: a cohort study. Am. J. Obstet. Gynecol..

[57] Kashani-ligumsky, L. et al. Titers of SARS CoV-2 antibodies in cord blood of neonates whose mothers contracted SARS CoV-2 (COVID19) during pregnancy and in those whose mothers were vaccinated with mRNA to SARS CoV-2 during pregnancy. *J. Perinatol.*10.1038/s41372-021-01216-1 (2021). 10.1038/s41372-021-01216-1 PMC8475451 34564695

[58] Shook LL (2022). Durability of anti-spike antibodies in infants after maternal COVID-19 vaccination or natural infection. JAMA.

[59] Flannery DD (2021). Assessment of maternal and neonatal cord blood SARS-CoV-2 antibodies and placental transfer ratios. JAMA Pediatr..

[60] Narayanaswamy V (2022). Neutralizing antibodies and cytokines in breast milk after coronavirus disease 2019 (COVID-19) mRNA vaccination. Obstet. Gynecol..

[61] Reza N. et al. mRNA COVID19 vaccines in pregnancy: a systematic review. *PLoS One*10.1371/journal.pone.0261350 (2022).

[62] Pratam, N. R. et al. COVID19 vaccination in pregnancy: a systematic review. *PLoS One*10.1371/journal.pone.0261350 (2021).

[63] Fu, W. et al. Systematic review of the safety, immunogenicity, and effectiveness of COVID-19 vaccines in pregnant and lactating individuals and their infants. *Int. J. Gynaecol. Obstet.*10.1002/ijgo.14008 (2022). 10.1002/ijgo.14008 PMC9087489 34735722

[64] Ma, Y. et al. Effectiveness and safety of COVID19 vaccine among pregnant women in real-world studies: a systematic review and meta-analysis. *Vaccines* **10**, 246 (2022). 10.3390/vaccines10020246 PMC8879911 35214704

[65] Dick A (2022). Safety of third SARS-CoV-2 vaccine (booster dose) during pregnancy. Am. J. Obstet. Gynecol. MFM.

[66] Wake, A. D. The acceptance rate toward COVID19 vaccine in Africa: a systematic review and meta-analysis. *Glob. Pediatr. Health*10.1177/2333794X211048738 (2021). 10.1177/2333794X211048738 PMC8488505 34616860

[67] Tomasz, G. et al. The approach of pregnant women to vaccination based on a COVID19 systematic review. *Medicina (Kaunas)*. **57**, 977 (2021). 10.3390/medicina57090977 PMC8468958 34577900

[68] Korber, B. et al. Tracking changes in SARS-CoV-2 spike: evidence that D614G increases infectivity of the COVID- ll tracking changes in SARS-CoV-2 spike: evidence that D614G increases infectivity of the COVID19 virus. *Cell*. 10.1016/j.cell.2020.06.043 (2020). 10.1016/j.cell.2020.06.043 PMC7332439 32697968

[69] Bian L (2021). Effects of SARS-CoV-2 variants on vaccine efficacy and response strategies. Expert Rev. Vaccines.

[70] Starr, T. N. Complete map of SARS-CoV-2 RBD Mutations that Escape the Monoclonal Antibody LY-CoV555 and its Cocktail with LY-CoV016. *Cell Rep Med*. 10.1101/2021.02.17.431683 2021. 10.1016/j.xcrm.2021.100255 PMC8020059 33842902

[71] Ayouni, I. et al. Effective public health measures to mitigate the spread of COVID19: a systematic review. *BMC Public Health* **21**, 1015 (2021). 10.1186/s12889-021-11111-1 PMC8164261 34051769

[72] Moher D (2010). Preferred reporting items for systematic reviews and meta-analyses: the PRISMA statement. Int J. Surg..

[73] Higgins J. P., Green S. *Cochrane Handbook for Systematic Reviews of Interventions: Cochrane Book Series*. (John Wiley & Sons, Ltd., 2008).

[74] Brooke BS, Schwartz TA, Pawlik TM (2021). MOOSE reporting guidelines for meta-analyses of observational studies. JAMA Surg..

[75] Higgins, J. P. et al. eds. *Cochrane Handbook for Systematic Reviews of Interventions* (John Wiley & Sons, 2019).

[76] National Heart, Lung, and Blood Institute. *Study Quality Assessment Tools.* https://www.nhlbi.nih.gov/health-topics/study-quality-assessment-tools (2021).

[77] Higgins J. P. T., Green S. *Cochrane Handbook for Systematic Reviews of Interventions: Cochrane Book Series*. (Wiley-Blackwell, 2008).

