# Supplementary material for: Maternal and neonatal outcomes of COVID-19 vaccination during pregnancy, a systematic review and meta-analysis
Source: NPJ Vaccines. 2023 Jul 15;8:103. doi: 10.1038/s41541-023-00698-8 (PMC10349851; doi:10.1038/s41541-023-00698-8)

Maternal and Neonatal Outcomes of COVID-19 Vaccination During Pregnancy, a Systematic Review and Meta-Analysis

Supplementary Files Legend

Supplementary Table Legend
Supplementary Table 1. Baseline characteristics of the included studies (1 of 2).
Supplementary Table 2. Baseline characteristics of the included studies (2 of 2).
Supplementary Table 3. Summary of the results of the quality assessment using the NHLB tool.
Supplementary Table 4. Pooled odds ratios from meta-analyses of studies exploring the associations of maternal, neonatal, and immunological outcomes with vaccination status in pregnant women.
Supplementary Table 5. Summary of maternal and umbilical cord blood antibody titers (N=19 studies).

Supplementary Figure Legend
Supplementary Figure 1. Forest plot of the odds ratio of non-operative (unassisted) vaginal delivery in vaccinated pregnant women vs. unvaccinated pregnant women using Mantel-Haenszel.
Supplementary Figure 2. Forest plot of the odds ratio of operative vaginal delivery in vaccinated pregnant women vs. unvaccinated pregnant women using Mantel-Haenszel.
Supplementary Figure 3. Forest plot of the odds ratio of gestational diabetes in vaccinated pregnant women vs. unvaccinated pregnant women using Mantel-Haenszel.
Supplementary Figure 4. Forest plot of the odds ratio of gestational hypertension in vaccinated pregnant women vs. unvaccinated pregnant women using Mantel-Haenszel.
Supplementary Figure 5. Forest plot of the odds ratio of placental abruption in vaccinated pregnant women vs. unvaccinated pregnant women using Mantel-Haenszel.
Supplementary Figure 6. Forest plot of the odds ratio of Postpartum Hemorrhage in vaccinated pregnant women vs. unvaccinated pregnant women using Mantel-Haenszel.
Supplementary Figure 7. Forest plot of the length of hospital stay at the time of delivery in vaccinated pregnant women vs. unvaccinated pregnant women using Inverse Variance. (A. Main meta-analysis, B. Meta-analysis after the exclusion of study contributing to heterogeneity).
Supplementary Figure 8. Forest plot of the preeclampsia in vaccinated pregnant women vs. unvaccinated pregnant women using Mantel-Haenszel.
Supplementary Figure 9. Forest plot of five minute Apgar score less than 7 in vaccinated pregnant women vs. unvaccinated pregnant women using Mantel-Haenszel.
Supplementary Figure 10. Forest plot of first-trimester miscarriage in vaccinated pregnant women vs. unvaccinated pregnant women using Mantel-Haenszel.
Supplementary Figure 11. Forest plot of fetal abnormalities in vaccinated pregnant women vs. unvaccinated pregnant women using Mantel-Haenszel.
Supplementary Figure 12. Forest plot of neonatal intensive care unit admission in vaccinated pregnant women vs. unvaccinated pregnant women using Mantel-Haenszel.
Supplementary Figure 13. Forest plot of small for gestational age in vaccinated pregnant women vs. unvaccinated pregnant women using Mantel-Haenszel.
Supplementary Figure 14. Forest plot of intrauterine growth restriction in vaccinated pregnant women vs. unvaccinated pregnant women using Mantel-Haenszel.
Supplementary Figure 15. Forest plot of stillbirth in vaccinated pregnant women vs. unvaccinated pregnant women using Mantel-Haenszel.

Supplementary Table 1 - Baseline Characteristics of the included studies (1 of 2)

Study (Author, year)	Country	Study design	Population description		Type of vaccination administered (Chemical name or brand name) (Proportion of participants administered)	Number of vaccination doses administere d	Vaccination -birth interval (days)	Vaccination time
			Cases	Control				
Ofer Beharier *et al.*, 2021	Israel	Retrospective cohort study	**Time period**: January-March 2021<br><br>**Enrollment**: At the time of delivery<br><br>**Number of vaccination doses administered**: one or two<br><br>**Cases:** Vaccinated pregnant women	**Time period**: April 2020-March 2021.<br><br>**Enrollment**: At the time of delivery<br><br>**Control group 1**: Matched unvaccinated pregnant women who became infected during pregnancy<br><br>**Control group 2**: Matched non-vaccinated and non-infected pregnant women	BNT162b2 mNRA vaccine/Pfizer (100%)	One or two doses	NR	During Pregnancy
Blakeway et al., 2022	United Kingdom	Retrospective cohort study	**Time period**: March 1st 2021-July 4th 2021<br>**Enrollment:** After delivery<br>**Inclusion criteria**:<br>Pregnant women with complete outcome data.<br>**Number of vaccination doses administered:** At least one dose<br><br>**Exclusion criteria**:<br>Women vaccinated before pregnancy or after labor<br>Women with genetic pregnancy complications.<br><br>Cases: Vaccinated pregnant women<br>Controls: Non-vaccination non-infected		Pfizer-BioNTech (77.8%)<br>Moderna (12.9%)<br>Oxford-AstraZeneca (9.3%)	At least one dose. (26 (18.6%) had 2 doses during pregnancy)	32.3 (20.2 - 53.4), median (IQR)	2nd trimester: 20 (14.3%)<br>3rd trimester: 120 (85.7%)

Inna Bleicher et al., 2021	Israel	Prospective cohort study	**Inclusion criteria**: Being pregnant when filling out the first questionnaire.		NR	78 (38.6%) received one dose<br>124 (61.4%) received two doses	NR	First questionnaire<br>1st trimester: 24 (30%)<br>2nd trimester: 37 (46%)<br>3rd trimester: 18 (24%)<br>Second questionnaire<br>1st trimester: 36 (17.8%)<br>2nd trimester: 110 (54.5%)<br>3rd trimester: 56 (27.7%)
Butt et al., 2021	Qatar	Retrospective cohort study	**Inclusion criteria**: Absence of COVID-19 infection within 14 days after the second vaccination dose.	**Inclusion criteria**:<br>**Group 1:**<br>Non-vaccinated pregnant women without any history of COVID-19 infection	NR	Two doses		First dose time<br>1st trimester: 323 (79.4%)<br>2nd trimester: 84 (20.6%)<br>The second dose was 14 days after the first.
Collier et al., 2021	Israel	Prospective Cohort study	**Time period**: December 2020-March 2021<br>**Age**: 18 years or older<br><br>**Number of vaccine doses administered**: Two	**Time period**: April 2020-March 2021<br><br>**Inclusion criteria**: COVID-19 Infected women	BNT162b2/Pfizer-BioNTech<br><br>mNRA-1273/Moderna	Two doses	NR	During pregnancy at any time throughout the three trimesters.
Dagan et al., 2021	Israel	Cohort study	Pregnant women aged 16 years or older, with no history of COVID-19, who were vaccinated between 20 December 2020 and 3 June 2021.	Matched unvaccinated pregnant controls.	BNT162b2 mNRA vaccine	NR	NR	During pregnancy at any time throughout the three trimesters.
Goldshtein et al., 2021	Israel	Retrospective cohort study	Pregnant women from December 19, 2020 (initiation of the national vaccination campaign), to	Matched newly pregnant unvaccinated women that had no prior records	BNT162b2 mNRA vaccine	NR	NR	During pregnancy at any time throughout the three trimesters.

			February 28, 2021. Excluded women who were infected with COVID-19 in the last year or who were vaccinated before pregnancy.	indicating a COVID-19 infection.				
Lipkind et al., 2022	United States	Retrospective cohort study	COVID-19 vaccination (a receipt of ≥1 COVID-19 vaccine doses) during pregnancy.	Matched unvaccinated pregnant women.	Pfizer-BioNTech, Moderna, Janssen (Johnson & Johnson)	At least one dose	NR	During pregnancy at any time throughout the three trimesters.
Magnus et al., 2021	Norway	Case-control study	All women vaccinated and non-vaccinated who were registered between February 15, 2021 and August 15, 2021, as having had a miscarriage before 14 weeks of gestation (case-patients) and those with a primary confirmation of ongoing pregnancy in the first trimester (controls).	Non-vaccinated women.	NR	NR	NR	First trimester
Morgan et al., 2022	United States	Retrospective cohort study	Patients were fully vaccinated two weeks before the start of the study period.	Control group of non-vaccinated women.	Pfizer = 833/ Moderna = 382/ Jassen = 67	NR	NR	During Pregnancy
Rottenstreich et al., 2021	Israel	Retrospective cohort study	All women aged 18 years or older, with no documented previous positive COVID-19 polymerase chain reaction test, delivered between 19 January 2021 (when the first vaccinated women gave birth) and 27 April 2021.		Pfizer– BioNTech BNT162b2	One or two doses	NR	3rd trimester
Shanes et al., 2021	United States	Cohort	Patients who tested negative for	Matched non-vaccinated	NR	NR	45.9 (24.3) total of 75	During Pregnancy

			COVID-19 infection and received the vaccine (delivering between January and April 2021).	non-infected pregnant women.				
Theiler et al., 2021	United States	Retrospective cohort study	All pregnant aged 16 to 55 years with a delivery event between December 10, 2020, and April 19, 2021, at a hospital within the Mayo Clinic Health System. Vaccinated individuals were defined as those receiving any dose of vaccine during pregnancy. For purposes of assessing vaccine effectiveness, fully vaccinated was defined as >14 days after the final dosage of vaccine.		Jassen 1 / Moderna 12/ Pfizer 127	NR	NR	During pregnancy
Wainstock et al., 2021	Israel	Retrospective cohort study	All women who delivered singleton pregnancies between January and June 2021 at the Soroka University Medical Center (SUMC) [15]. Excluded from the study were women diagnosed with COVID-19 in the past, women with multiple gestations or women with unknown vaccination status or incomplete pregnancy follow-up information.		NR	One or two doses	NR	During 2nd or 3rd trimesters
Shimabukuro et al., 2021	United States	Retrospective cohort study	Enrolled patients on V-safe surveillance system or VAERS national reporting system who received vaccination during pregnancy or in the periconceptional period (30 days before the last menstrual period through 14 days after) and were 18 years of age or older.	No control	V-safe:<br>Pfizer-BioNTech vaccine = (14,320)<br>Moderna vaccine = (13,232)<br><br>VAERS:<br>Pfizer-BioNTech = 130 (58.8%)<br>Moderna = 90 (40.7%)	One or two doses	NR	Total patients in V-safe:<br>- Pregnant at time of vaccination = 30,887 (86.5%)<br>- Positive pregnancy test after vaccination = 4,804 (13.5%)<br><br>Patients were followed for pregnancy neonatal outcomes in V-safe:<br>- Periconception vaccination = 92 (2.3%)<br>- First trimester: <14 wk = 1132 (28.6%)<br>- Second trimester: ≥14 and <28 wk = 1714 (43.3%)<br>- Third trimester: ≥28 wk

								= 1019 (25.7%)<br><br>VAERS patients: n =163<br>- 1st trimester = 81 (49.7%)<br>- 2nd trimester = 53 (32.5%)<br>- 3rd trimester = 29 (17.8%)
†Caroline Atyeo et al., 2021	United States	Prospective cohort study	Enrolled women from two tertiary centers between December 17th 2020 and February 23rd 2021: 84 pregnant, 31 lactating women, and 16 nonpregnant. All the patients were at the reproductive age from 18 to 45	Matched vaccinated non-pregnant women (16) and vaccinated lactating women (31).	BNT162b2 (Pfizer) 65<br>mNRA-1273 (Moderna) 66	NR	NR	During pregnancy
Bashi et al., 2021	Israel	Prospective cohort study	Pregnant women aged 18 to 45 who delivered at a University Tertiary Medical Center from December 2020 to March 2021 and received the Pfizer vaccine during pregnancy.<br>Women were excluded if they had previous COVID-19 infection, refused to sign the informed consent, or lacked a verified time of pregnancy.	NR	BNT162b2 (Pfizer)	Nineteen women received one dose and<br>39 received two doses	1st dose median (IQR) = 30 (19.7 - 40.5)<br>2nd dose = 15 (8-23)	During pregnancy
Bookstein Peretz et al., 2021	Israel	Case-control	Pregnant women who received two doses of the Pfizer vaccination during pregnancy at 2 - 40 weeks of gestation between January and February 2021 and did	Matched with non-pregnant women receiving 2 doses of the vaccine.	BNT162b2 (Pfizer)	Two doses of vaccine	NR	During pregnancy<br>1st dose<br>76 in 1st trimester<br>193 in 2nd trimester<br>121 in 3rd trimester<br>2nd dose<br>52 in 1st trimester

			not deliver before the second dose.					154 in 2nd trimester<br>184 in 3rd trimester
Dawood et al., 2021	United States	Prospective cohort study	Pregnant women at <28 weeks of gestation aged between 18 and 50 years old, who were capable of self collecting and mailing swab specimens weekly, and agreed to share neonatal history, and were enrolled in COVID-19 or influenza vaccine clinical trials. Of a total of 1098 eligible women, only 400 had available information about COVID-19 vaccines during pregnancy and completed the postpartum follow-up period: 91 women were vaccinated and 309 were unvaccinated.		Fully vaccinated = 66<br>Partially vaccinated = 25	NR	NR	During pregnancy
†Gray et al., 2021	United States	Prospective cohort study	Enrolled women from two tertiary centers between December 17th 2020 and February 23rd 2021: 84 pregnant, 31 lactating women, and 16 nonpregnant. All the patients were at the reproductive age from 18 to 45 and received COVID-19 vaccines as BNT162b2 and mNRA-1273.	Matched vaccinated non-pregnant women (16), vaccinated lactating women (31), and infected pregnant women (37).	Pfizer-BioNTech = 65<br>Moderna = 66	At least one dose	Median (IQR) of 13 delivered patients<br>1st dose = 36.5 (30-42)<br>2nd dose = 14 (11-16)	During pregnancy<br>- First trimester = 11 (13%)<br>- Second trimester = 39 (46%)<br>- Third trimester = 34 (40%)
Hillson et al., 2021	United Kingdom<br>South Africa<br>Brazil	Prospective cohort study	Analysis of pregnancies that occurred in four clinical trials in three countries: United Kingdom (NCT04324606 and NCT04400838), South Africa (NCT04444674), and Brazil (NCT04536051). All participants were 49 years or younger and randomly allocated into two groups:	Unvaccinated control pregnant women who became pregnant during the trial	ChAdOx1 nCoV-19 vaccine	NR	From the first dose to pregnancy vaccine group = 126.7 ± 94.9<br>control group = 130.6 ± 93.6	Before pregnancy

			ChAdOx1 nCoV-19 and control group. All pregnant-vaccinated women received vaccines before pregnancy.					
Kachikis et al., 2021	United States	Prospective cohort study	Pregnant (7809), lactating (6815), or planning pregnancy (2901) at the time of COVID-19 vaccination were recruited online at the University of Washington		In all groups<br>Pfizer-BioNTech BNT162b2 = 10790<br>Moderna mNRA-1273 = 6529<br>Janssen JNJ-78436735 = 49	At least one dose	NR	In pregnancy (7611)<br>First trimester = 1822 (23.9%)<br>Second trimester = 3694 (48.5%)<br>Third trimester = 2095 (27.5%)
Kashani-Ligumsky et al., 2021	Israel	Cohort study	Vaccinated with two doses of the BNT162b2- mNRA SARS-CoV-2 Pfizer vaccine in the third trimester of pregnancy.	Twenty-nine women who were infected with COVID-19 during pregnancy. This included women who had a positive test during pregnancy or those found to have positive serology at delivery, and 21 women who were not vaccinated and had no evidence of SARS-CoV-2 infection during pregnancy	Pfizer	Two doses	NR	During pregnancy throughout the 3 trimesters
Kugelman et al., 2021	Israel	Cohort study	Pregnant women over 24 weeks of gestation expected to give birth within 3 days who had received their second dose Pfizer within 7 days and were not known to be previously infected.	No control	Pfizer	Two doses	Time from 2nd dose was a mean of 14.4 ±3 days.	Second trimester

Mithal et al., 2021	United States	Retrospective cohort study	Women who received a COVID-19 vaccination during pregnancy.	No control	18 (64%) received the Pfizer vaccine, 6 (18%) received the Moderna vaccine, and 4 (14%) received an unknown vaccine.	Two doses	Mean latency of 63 weeks.	During pregnancy
Nakahara et al., 2022	United States	Prospective cohort study	Women between ages 18-55 years with a documented active pregnancy who underwent the first of two rounds of immunization with either FDA-approved mNRA COVID-19 vaccine from December 14th 2020, through January 28th 2021.	1:2 with non-pregnant, age-matched female controls that also received the vaccine during the same period.	Pfizer & Moderna	Two doses		Starting dose mean 18.4 ± 9.2 weeks of gestation
Nir et al., 2022	Israel	Prospective cohort study	Pregnant women aged ≥18 years who had received two dosages of the BNT162b2 mNRA COVID-19 vaccine at least 14 days prior to delivery.	COVID-19 recovered women with previously documented positive polymerase chain reaction tests for COVID-19 served as a control group; patients were defined as recovered if they were asymptomatic for 3 days or more after 10 days had elapsed from the initial positive PCR test.	BNT162b2 mNRA COVID-19 vaccine	Two doses	At least 14 days before delivery	During pregnancy
Prabhu et al., 2021	United States	Cohort study	Women self-reported receipt of one or both doses of a messenger NRA (mNRA) based	No control.	Pfizer & Moderna	One or two doses	NR	3rd trimester

			COVID-19 vaccine and gave birth to a singleton neonate (gestational age between 35 0/7 and 41 2/7 weeks) were included.					
Prahl et al., 2021	United States	Cohort study	20 pregnant individuals who received COVID-19 mNRA vaccines during pregnancy & their infants.	No control.	Pfizer or Moderna	2 doses	1st dose range 6-97 (mean 51, SD 24.3), 2nd dose range 2-75 (M 32, SD 21.3) days prior to delivery, and in two participants 15 and 21 days after delivery.	Ranged from 13 weeks to 40 weeks (mean 31.2, SD 5.9 weeks)
Shen et al., 2022	Taiwan	Cohort study	All patients in the study had singleton pregnancies without symptoms related to COVID-19 during pregnancy. They had voluntarily vaccinated against COVID-19 with first dose administration between the 27th and 38th week of gestation. The exclusion criteria were age below 20 years, COVID-19 vaccination prior to pregnancy, preterm labor, and disease with immunosuppressant treatment.	No control.	Moderna	At least one dose	NR	3rd trimester

Trostle et al., 2021	United States	Retrospective cohort study	Pregnant women at New York University Langone Health who received at least one dose of an mNRA COVID-19 vaccination approved by the Food and Drug Administration (FDA) (Pfizer-BioNTech or Moderna) from the time of the FDA Emergency Use Authorization to April 22, 2021.	No control.	Pfizer and Moderna	At least 1 dose	2.86 (0.29−12.7) median (IQR)	During Pregnancy
Yang et al., 2021	United States	Retrospective cohort study	Pregnant women with a self-reported history of at least one dose of an mNRA-based COVID-19 vaccine (Pfizer-BioNTech BNT162b2 [Pfizer] or Moderna mNRA-1273 [Moderna]) or one dose of a viral vector–based COVID-19 vaccine (Johnson & Johnson/Janssen JNJ-78436735 [J&J/Janssen]) prior to or during pregnancy, and who delivered a liveborn neonate at 34 weeks of gestation or more at a single academic medical center between March 6, 2021 and October 28, 2021.	No control.	Pfizer, Moderna, or Janssen	at least 1 dose	NR	Before or during Pregnancy
Zauche et al., 2021	United States	Retrospective cohort study	Participants with a singleton pregnancy who received at least one preconceptional	No control.	Pfizer & Moderna	At least one dose	NR	Preconceptional (30 days before the first day of the last menstrual period through 14 days after) or

			dose of an mNRA COVID-19 vaccine (30 days before the first day of the last menstrual period through 14 days after) or one dose during pregnancy before 20 weeks gestation, and who had not reported a pregnancy loss before 6 completed weeks' gestation. Participants who were enrolled in the v-safe pregnancy registry who had received the Janssen vaccine (n = 272) were not included in this analysis.					during pregnancy before 20 weeks gestation
Zdanowski et al., 2021	Poland	Retrospective cohort study	Vaccinated with two doses of BNT162b2 mNRA COVID-19 vaccine between the 29th and 36th week of gestation; that is, the first dose was administered between the 29th and 36th week of gestation (the first dose) and the second dose between the 32nd and 40th week of pregnancy.	No control.	BNT162b2 mNRA COVID-19 vaccine	Two doses	NR	3rd trimester
Rottenstreich, A et al., April 2021	Israel	Cohort study	Women who received at least one dose of SARS-CoV-2 BNT162b2 mNRA vaccine.	No control.	BNT162b2 mNRA	Two doses	Median time from the first and second doses of vaccine was 33, IQR (30-37) and 11, IQR	During pregnancy

							(9-1) days, respectively.	
Rottenstreich, A et al., November 2021	Israel	Cohort study	Women who received the SARS-CoV-2 BNT162b2 mNRA vaccine during pregnancy were consecutively approached following their admission to the delivery room and offered participation.	No control.	BNT162b2 mNRA vaccine	Two doses	NR	3rd trimester
Matsui et al., 2021	United States	Cohort study	Pregnant individuals who received an mNRA-based COVID-19 vaccine were enrolled from December 2021.	30 infected pregnant women were chosen from these two cohorts to be matched on the approximate gestational age of first vaccine dose and the earliest confirmed SARS-CoV-2 test.	mNRA-1273 BNT162b2	One or two doses	91 days (60-140), median (IQR)	During pregnancy

†The two studies had the same population; however, they reported different outcomes, IQR = Interquartile Range, and NR = Not Reported

Supplementary Table 2 - Baseline Characteristics of the included studies (2 of 2)

Study name	Study groups	Maternal age (year) Mean (SD)	Gestational age (weeks) Mean (SD)	Maternal BMI (Kg/m2) Mean (SD)	Gravidity	Parity	Pre-gestational diabetes mellitus (events)	Hypertension (events)
Ofer Beharier et al., 2021	Vaccinated pregnant (n = 92)	31.7 (5.8)	39.3 (1.3)	24.2 (5.2)	median (IQR) 3 (2)	median (IQR) 1 (2)	8	1
	Non-vaccinated non-infected (n = 66)	31.6 (5.8)	39.2 (1.4)	25.7 (6.5)	median (IQR) 3 (2.5)	median (IQR) 2 (2)	9	1
	Non-vaccinated Infected (n = 74)	28.8 (5.8)	39 (1.6)	26.4 (9.2)	median (IQR) 3 (2)	median (IQR) 1 (3)	4	1
Blakeway et al., 2022	Vaccinated pregnant (n = 140)	34.5 (3.9)	NR	24.3 (4.5)	NR	N = 63	6	13
	Non-vaccinated non-infected (n = 1188)	33 (4.5)	NR	24.8 (4.8)	NR	N = 593	7	46
Inna Bleicher et al., 2021	Vaccinated pregnant (n = 80)	31.7 (3.9)	21.4 (9.9)	NR	NR	NR	NR	NR
	Non-vaccinated non-infected (n = 233)	30.2 (5.09)	21.9 (9.4)	NR	NR	NR	NR	NR
Butt et al., 2021	Vaccinated pregnant (n = 407)	32.3 (5.2)	<12 weeks = 63 (15.5%)<br>12 -15 weeks = 122 (30%)<br>16-19 weeks = 110 (27%)<br>20 - 24 weeks = 112 (19.8%)	NR	NR	NR	NR	NR
	Non-vaccinated non-infected (n = 407)	32 (5.95)	<12 weeks = 43 (10.6%)<br>12 -15 weeks = 89 (21.9%)<br>16-19 weeks = 140(34.4%)<br>20 - 24 weeks = 135 (33.2%)	NR	NR	NR	NR	NR
Collier et al., 2021	Vaccinated pregnant (n = 30)	35 (32-36)	NR	NR	NR	NR	NR	NR
	Non-vaccinated Infected pregnant (n = 22)	31(28-36)	NR	NR	NR	NR	NR	NR
Dagan et al., 2021	Vaccinated pregnant (n = 10861)	30 (26,33)	NR	BMI> 30 = 1148	NR	NR	71	40
	Non-vaccinated non-infected	30 (26,33)	NR	BMI> 30 = 1115	NR	NR	64	34

	(n = 10861)							
Goldshtein et al., 2021	Vaccinated pregnant (n = 7530)	31.1 (5.01)	NR	825 (11)	NR	NR	63	51
	Non-vaccinated non-infected (n = 7530)	31 (4.85)	NR	793 (10.5)	NR	NR	30	58
Lipkind et al.,2022	Vaccinated pregnant (n = 10064)	32.3 (4.5)	NR	2407 (23.9)	NR	NR	167	525
	Non-vaccinated non-infected (n = 36015)	29.8 (5.3)	NR	10426 (29)	NR	NR	611	1732
Magnus et al.,2021	Vaccinated pregnant (n = 1003)	30.72 (5.65)	NR	NR	NR	≥1 = 360	NR	NR
	Non-vaccinated non-infected (n = 17474)	31.19 (5.2)	NR	NR	NR	≥1 = 6773	NR	NR
Morgan et al.,2022	Vaccinated pregnant (n = 1332)	32.1 (5.9)	28.8 (7.32)	NR	NR	NR	22	79
	Non-vaccinated non-infected (n = 8760)	27.8 (4.9)	30.4 (8.1)	NR	NR	NR	180	614
Rottenstreich M et al., 2021	Vaccinated pregnant (n = 712)	30.6 (5.8)	39.1 (1.6)	BMI> 30 =101	median (IQR) 4 (2-6)	media n (IQR) 3 (2-6)	NR	NR
	Non-vaccinated non-infected (n = 1063)	29.5 (6)	39.4 (1.6)	BMI> 30 =140	median (IQR) 4 (2-6)	media n (IQR) 3 (2-5)	NR	NR
Shanes et al., 2021	Vaccinated pregnant (n = 84)	33.7 (3.1)	38.5 (2.4)	NR	NR	NR	NR	NR
	Non-vaccinated non-infected (n = 116)	32.5 (4.8)	38.4 ((1.9)	NR	NR	NR	NR	NR
Theiler et al., 2021	Vaccinated pregnant (n = 140)	31.8 (3.7)	37 weeks = 127<br>32 - 36 weeks = 10<br>24 - 31 weeks = 2<br>24 weeks = 1	BMI> 30 = 33	1=56<br>2=34<br>3=29<br>≥4 =21	NR	2	6
	Non-vaccinated non-infected (n = 1862)	30.5 (5.2)	37 weeks = 1703<br>32 - 36 week = 134<br>24 - 31 week = 21<br>24 week = 4	BMI > 30 = 464	1=546<br>2=519<br>3=350<br>≥4 =447	NR	11	64
Wainstock et al., 2021	Vaccinated pregnant (n = 913)	30.6 (5.3)	38.9 (1.4)	BMI > 30 = 152	NR	NR	NR	NR
	Non-vaccinated non-infected (n =3486)	28.2 (5.7)	39	BMI > 30 = 549	NR	NR	NR	NR

Shimabukuro et al., 2021	Vaccinated pregnant in V-safe (n = 3958)	NR	20–24 year = 36 (0.9%)<br>25–34 year = 2573 (65.0%)<br>35–44 year = 1337 (33.8%)<br>45–54 year = 12 (0.3%)	NR	NR	NR	NR	NR
	Vaccinated pregnant in VERS (n = 163)	33.1 ± 26.1	NR	NR	NR	NR	NR	NR
†Caroline Atyeo et al., 2021	Vaccinated pregnant (n = 84)	NR	NR	NR	NR	NR	NR	NR
	Non-vaccinated Infected pregnant (n = 37)	NR	NR	NR	NR	NR	NR	NR
Bashi et al., 2021	Vaccinated pregnant (n = 58)	32.3 (2.8)	at pregnancy = 38.73 (0.91)<br>at 1st dose = 34.5 (2.28)<br>at 2nd dose = 37 (1.5)	22.3 (3.04)	NR	Null parity = 25	NR	NR
Bookstein Peretz et al., 2021	Vaccinated pregnant (n = 390)	32.5 (3.7)	Total = 57<br>39.4 (0.98)	Total = 390<br>24.4 (5.2)	NR	NR	Total = 390<br>N = 13	Total = 390<br>N = 2
Dawood et al., 2021	Vaccinated pregnant (n = 1098)	30 (5.9)	at enrollment (0 to 13 weeks) = 366 (33%)<br>(14 to 27 weeks) = 732 (67%)	NR	NR	Primipara = 327	NR	NR
†Gray et al., 2021	Vaccinated pregnant (n = 84)	34.1 (3.3)	At first vaccine dose = 23.68 (11.91)<br>at delivery (n= 13) = 39.53 (1.07)	NR	NR	NR	3	3
	Non-vaccinated Infected pregnant (n = 37)	32.5 (5.3)	Infected pregnant women at COVID19 diagnosis = 30.1 (26.9 - 33.8) median (IQR)	NR	median (IQR)<br>2 (2-3)	median (IQR)<br>1 (0-1)	NR	NR

Hillson et al., 2021	Vaccinated pregnant (n = 72)	31.6 (5.9)	NR	26.4 (5.8)	NR	NR	NR	NR
	Non-vaccinated non-infected (n = 35)	29.2 (6.5)	NR	25.9 (5.5)	NR	NR	NR	NR
Kachikis et al., 2021	Vaccinated pregnant (n = 7804)	33.4 (3.6)	NR	NR	Total = 5236 2.1 (1.3) Mean (SD)	Total = 5245 1.2 (1.0) Mean (SD)	NR	NR
Kashani-Ligumsky et al., 2021	Vaccinated pregnant (n = 29)	32.5	39.3	NR	NR	Mean = 5.2	NR	NR
	Non-vaccinated non-infected (n = 21)	28.5	38.8	NR	NR	Mean = 2.7	NR	NR
Kugelman et al., 2021	Vaccinated pregnant (n = 130)	31.9 (4.9)	39.3 (1.3)	median IQR 27.5 (24.9-30)	NR	32	NR	NR
Mithal et al., 2021	Vaccinated pregnant (n = 27)	33 (3)	NR	NR	NR	NR	NR	NR
Nakahara et al., 2022	Vaccinated pregnant (n = 83)	32 (3.9)	NR	26.1 (5.9)	Mean (SD) = 2 (1.2)	Mean (SD) = 0.6 (0.8)	NR	NR
Nir et al., 2022	Vaccinated pregnant (n = 64)	33.8 (5.8)	38.7 (1.3)	28.2 (4.9)	NR	NR	1	1
	Non-vaccinated Infected pregnant (n = 11)	32.7 (5.7)	39 (1.2)	31.6 (4.6)	NR	NR	1	0
Prabhu et al., 2021	Vaccinated pregnant (n = 122)	NR	between 35 0/7 and 41 2/7 weeks)	NR	NR	NR	NR	NR
Prahl et al., 2021	Vaccinated pregnant (n = 20)	34.55 (2.89)	39.2 (1.1)	NR	NR	NR	NR	NR
Shen et al., 2022	Vaccinated pregnant (n = 29)	33.21 (3.89)	38.55 (1.12)	Median (IQR) 27.42 (29.6-24.3)	NR	Median (IQR) 20 (1-0)	NR	NR
Trostle et al., 2021	Vaccinated pregnant (n = 424)	35 (6)	Median (IQR) 39.3 (33-41.7)	23.2 (5.2)	NR	NR	5	28
Yang et al., 2021	Vaccinated pregnant (n = 1321)	35.31 (3.755)	39.32 (0.65)	NR	Median (IQR) = 2 (0-4)	Median (IQR) = 0 (0-1)	NR	NR
Zauche et al., 2021	Vaccinated pregnant (n = 2456)	events: (20-29 year) = 432, (30-34 year) = 1205, (35-39 year) = 693,	NR	BMI > 30 = 432	NR	NR	27	NR

		(+40 year) = 126						
Zdanowski et al., 2021	Vaccinated pregnant (n = 16)	31.2 (2.2)	39.69 (1.01)	BMI > 30 = 11	NR	NR	NR	NR
Rottenstreich, A et al., April 2021	Vaccinated pregnant (n = 20)	Median (IQR) = 32 (28-37)	Median (IQR) = 39 (38-40)		NR	NR	NR	NR
Rottenstreich, A et al., November 2021	Vaccinated pregnant (n = 171)	31.37 (5.2)	Median (IQR) = 39 (38-40)	Median (IQR) = 28 (26-31)	NR	Mean (SD) = 2.8 (2.25)	NR	NR
Matsui et al., 2021	Vaccinated pregnant (n = 30)	Median (IQR) = 36 (33-38)	Median (IQR) = 39.1 (37.7-39.6)	Median (IQR) = 23.5 (22-27.3)	NR	NR	NR	NR
	Non-vaccinated non-infected (n = 4)	Median (IQR) = 32 (28-35)	Median (IQR) = 38 (37.5-40)	NR	NR	NR	NR	NR
	Non-vaccinated Infected pregnant (n = 30)	Median (IQR) = 28 (24-36)	Median (IQR) = 39.1 (38.7-39.3)	Median (IQR) = 32.2 (28.8-35.9)	NR	NR	NR	NR

†The two studies had the same population; however, they reported on different outcomes, BMI = Body Mass Index, IQR = Interquartile Range, and NR = Not Reported

Supplementary Table . Results of the quality assessment of the included studies.

Study ID/Domain	Q1. Was the research question or objective in this paper clearly stated?	Q2. Was the study population clearly specified and defined?	Q3. Was the participation rate of eligible persons at least 50%?	Q4. Were all the subjects selected or recruited from the same or similar populations?	Q5. Was a sample size justification, power description, or variance and effect estimates provided?	Q6. For the analyses in this paper, were the exposure(s) of interest measured prior to the outcome(s) being measured?	Q7. Was the timeframe sufficient so that one could reasonably expect to see an association between exposure and outcome if it existed?	Q8. For exposures that can vary in amount or level, did the study examine different levels of the exposure?
Caroline Atyeo et al, 2021	Yes	Yes	NA	Yes	No	NA	Yes	NA
Bashi et al, 2021	Yes	Yes	NA	Yes	No	NA	Yes	NA
Beharier et al, 2021	Yes	Yes	Yes	Yes	No	NA	Yes	NA
Blakeway et al, 2022	Yes	Yes	Yes	Yes	No	NA	Yes	NA
Bleicher et al, 2021	Yes	Yes	Yes	Yes	Yes	NA	Yes	NA
Bookstein Peretz et al, 2021	Yes	Yes	Yes	Yes	No	NA	Yes	NA
Butt et al, 2021	Yes	Yes	Yes	Yes	No	NA	Yes	NA
Collier et al, 2021	Yes	Yes	Yes	Yes	No	NA	Yes	NA
Dagon et al, 2021	Yes	Yes	Yes	Yes	No	NA	Yes	NA
Dawood et al, 2021	Yes	Yes	No	Yes	Yes	NA	Yes	NA
Goldshtein et al, 2021	Yes	Yes	Yes	Yes	No	NA	Yes	NA
Gray et al, 2021	Yes	Yes	NA	Yes	No	NA	Yes	NA
Hillson et al, 2021	Yes	Yes	NA	Yes	No	NA	Yes	NA
Kachikis et al, 2021	Yes	Yes	NA	Yes	No	NA	Yes	NA
Kashani-Ligumsky et al, 2021	Yes	Yes	NA	Yes	No	NA	Yes	NA
Kugelman et al, 2021	Yes	Yes	NA	Yes	No	NA	Yes	NA
Lipkind et al,2022	Yes	Yes	Yes	Yes	No	NA	Yes	NA
Magnus et al, 2021	Yes	Yes	NA	Yes	No	NA	Yes	NA
Mithal et al, 2021	Yes	Yes	NA	Yes	No	NA	Yes	NA

Morgan et al, 2022	Yes	Yes	NA	Yes	No	NA	Yes	NA
Nakahara et al, 2022	Yes	Yes	NA	Yes	No	NA	Yes	NA
Nir et al, 2022	Yes	Yes	NA	Yes	No	NA	Yes	NA
Prabhu et al, 2021	Yes	Yes	NA	Yes	No	NA	Yes	NA
Prahl et al, 2021	Yes	Yes	NA	Yes	No	NA	Yes	NA
Rottenstreich M et al, 2021	Yes	Yes	Yes	Yes	No	NA	Yes	NA
Shanes et al, 2021	Yes	Yes	NA	Yes	No	NA	Yes	NA
Shen et al, 2022	Yes	Yes	NA	Yes	No	NA	Yes	NA
Shimabukuro et al, 2021	Yes	Yes	NA	Yes	No	NA	Yes	NA
Theiler et al, 2021	Yes	Yes	NA	Yes	No	NA	Yes	NA
Trostle et al, 2021	Yes	Yes	NA	Yes	No	NA	Yes	NA
Wainstock et al, 2021	Yes	Yes	Yes	Yes	No	NA	Yes	NA
Yang et al, 2021	Yes	Yes	NA	Yes	No	NA	Yes	NA
Zauche et al, 2021	Yes	Yes	NA	Yes	No	NA	Yes	NA
ZdaNowski et al, 2021	Yes	Yes	NA	Yes	No	NA	Yes	NA
Rottenstreich et al, A et al, 2021a	Yes	Yes	NA	Yes	No	NA	Yes	NA
Rottenstreich et al, A et al, 2021b	Yes	Yes	Yes	Yes	No	NA	Yes	NA
Mastui et al, 2021	Yes	Yes	NA	Yes	No	NA	Yes	NA

Study ID/Domain	Q9. Were the exposure measures (independent variables) clearly defined, valid, reliable, and implemented consistently across all study participants?	Q10. Was the exposure(s) assessed more than once over time?	Q11. Were the outcome measures (dependent variables) clearly defined, valid, reliable, and implemented? consistently across all study participants?	Q12. Were the outcome assessors blinded to the exposure status of participants?	Q13. Was loss to follow-up after baseline 20% or less	Q14. Were key potential confounding variables measured and adjusted statistically for their impact on the relationship? between exposure(s) and outcome(s)?	**Summary Quality**
Caroline Atyeo et al, 2021	Yes	No	Yes	NA	NA	No	**8**
Bashi et al, 2021	Yes	No	Yes	NA	Yes	Yes	**10**
Beharier et al, 2021	Yes	No	Yes	Yes	Yes	Yes	**10**
Blakeway et al, 2022	Yes	No	Yes	NA	Yes	Yes	**9**
Bleicher et al, 2021	Yes	No	Yes	No	Yes	No	**9**
Bookstein Peretz et al, 2021	Yes	No	Yes	NA	Yes	No	**10**
Butt et al, 2021	Yes	No	Yes	NA	Yes	No	**8**
Collier et al, 2021	Yes	No	Yes	NA	Yes	No	**8**
Dagon et al, 2021	Yes	No	Yes	NA	Yes	No	**8**
Dawood et al, 2021	Yes	No	Yes	NA	Yes	No	**9**
Goldshtein et al, 2021	Yes	No	Yes	NA	Yes	No	**8**
Gray et al, 2021	Yes	No	Yes	NA	Yes	No	**9**
Hillson et al, 2021	Yes	No	Yes	NA	Yes	No	**9**
Kachikis et al, 2021	Yes	No	Yes	NA	Yes	No	**9**
Kashani-Ligumsky et al, 2021	Yes	No	Yes	NA	Yes	No	**9**
Kugelman et al, 2021	Yes	No	Yes	NA	Yes	Yes	**10**
Lipkind et al,2022	Yes	No	Yes	NA	Yes	No	**8**
Magnus et al, 2021	Yes	No	Yes	NA	Yes	No	**7**
Mithal et al, 2021	Yes	No	Yes	NA	Yes	Yes	**10**
Morgan et al, 2022	Yes	No	Yes	NA	Yes	No	**7**
Nakahara et al, 2022	Yes	No	Yes	NA	Yes	No	**9**

Nir et al, 2022	Yes	No	Yes	NA	Yes	Yes	**10**
Prabhu et al, 2021	Yes	No	Yes	NA	Yes	Yes	**10**
Prahl et al, 2021	Yes	No	Yes	NA	Yes	Yes	**10**
Rottenstreich M et al, 2021	Yes	No	Yes	NA	Yes	Yes	**9**
Shanes et al, 2021	Yes	No	Yes	NA	Yes	No	**7**
Shen et al, 2022	Yes	No	Yes	NA	Yes	Yes	**10**
Shimabukuro et al, 2021	Yes	No	Yes	NA	Yes	No	**9**
Theiler et al, 2021	Yes	No	Yes	NA	Yes	No	**7**
Trostle et al, 2021	Yes	No	Yes	NA	Yes	No	**9**
Wainstock et al, 2021	Yes	No	Yes	NA	Yes	No	**8**
Yang et al, 2021	Yes	No	Yes	NA	Yes	Yes	**10**
Zauche et al, 2021	Yes	No	Yes	NA	Yes	No	**9**
ZdaNowski et al, 2021	Yes	No	Yes	NA	Yes	Yes	**10**
Rottenstreich et al, A et al, 2021a	Yes	No	Yes	NA	Yes	No	**7**
Rottenstreich et al, A et alb	Yes	No	Yes	NA	Yes	No	**8**
Mastui et al, 2021	Yes	No	Yes	NA	Yes	No	**7**

Supplementary Table 4. Pooled odds ratios from meta-analyses of double arm studies exploring the associations of maternal, neonatal, and immunological outcomes with vaccination status in pregnant women.

**Outcome**	**No. of studies**	**References**	**Vaccinated pregnant women n (%)**			**Non-vaccinated pregnant women n (%)**			**OR (95%CI)**	**P**	**I^2 (%)**	**P_{het}**	***Model***
			Outcome Yes	Outcome No	Total	Outcome Yes	Outcome No	Total					
Maternal outcomes													
**Unassisted vaginal delivery**	4	Blakeway et al., 2022, Kashani-Ligumsky et al., 2021, Rottenstreich Met al., 2021, Theiler et al., 2021	197 (19.5)	817 (80.5)	1014 (23.2)	1496 (44.7)	1849 (55.3)	3345 (77.8)	0.90 (0.71-1.16)	0,42	0	0.95	F
**Operative vaginal delivery**	4	Blakeway et al. 2022, Rottenstreich M et al., 2021, Theiler et al., 2021, Wainstock et al. 2021	79 (4.1)	1819 (95.8)	1898 (21.8)	311 (4.5)	6499 (95.4)	6810 (78.2)	0.93 (0.55-1.55)	0.77	72	0.01	R
**Cesarean section**	4	Blakeway et al. 2022, Rottenstreich M et al., 2021, Theiler et al., 2021, Wainstock et al. 2021	378 (19.9)	1520 (80.0)	1898 (21.8)	1407 (20.6)	5403 (79.3)	6810 (78.2)	1.20 (1.05-1.38)	0.007[a]	45	0.14	F
**Gestational diabetes**	2	Blakeway et al., 2022, Wainstock et al. 2021	83 (7.8)	970 (92.1)	1053 (18.3)	333 (7.1)	4341 (92.8)	4674 (81.6)	1.28 (0.99-1.65)	0.06	0	0.75	F
**Gestational hypertension**	2	Theiler et al. 2021, Wainstock et al. 2021	69 (6.5)	984 (93.4)	1053 (16.4)	390 (7.2)	4958 (92.7)	5348 (83.5)	1.16 (0.88-1.52)	0.29	0	0.95	F
**Preeclampsia**	2	Goldshtein et al. 2021, Theiler et al. 2021	21 (0.2)	7649 (99.7)	7670 (44.9)	44 (0.4)	9348 (99.5)	9392 (55.0)	0.90 (0.50-1.61)	0.73	0	0.64	F
**Placental abruption**	3	Blakeway et al. 2022, Rottenstreich M et al., 2021, Wainstock et al. 2021	11 (0.6)	1747 (99.3)	1758 (26.1)	36 (0.7)	4922 (99.2)	4958 (73.8)	0.58 (0.29-1.14)	0.11	6	0.30	F
**Postpartum hemorrhage**	4	Blakeway et al. 2022, Rottenstreich M et al., 2021, Theiler et al., 2021, Wainstock et al. 2021	76 (4.0)	1822 (96.0)	1898 (21.8)	177 (2.6)	6633 (97.4)	6810 (78.2)	0.85 (0.64-1.12)	0.24	23	0.28	F
**Length of hospital stay (at the time of delivery)**	3	Rottenstreich M et al., 2021, Theiler et al., 2021, Wainstock et al. 2021	NA	NA	1765	NA	NA	6411	-0.40 (-0.88-0.08) *	0.10	94	<0.0 0001	R
Neonatal outcomes													
**Preterm birth**	3	Goldshtein et al. 2021, Lipkind et al. 2022, Theiler et al. 2021	580 (4.8)	11011 (95.1)	11591 (22.7)	2740 (6.9)	36564 (93.0)	39304 (77.2)	0.71 (0.64-0.78)	<0.000 01[a]	53	0.12	F
**5-minute Apgar score <= 7**	3	Rottenstreich M et al., 2021, Theiler et al., 2021, Wainstock et al. 2021	26 (1.4)	1739 (98.5)	1765 (21.5)	95 (1.4)	6316 (98.5)	6411 (78.4)	0.86 (0.54-1.37)	0.52	50	0.14	F

**First trimester miscarriage**	2	Goldshtein et al. 2021, Magnus et al. 2021	359 (4.2)	8174 (95.7)	8533 (25.4)	4408 (17.6)	20596 (82.3)	25004 (74.5)	0.96 (0.84-1.09)	0.54	19	0.27	F
**Fetal abnormalities**	2	Blakeway et al. 2022, Bleicher et al. 2021	12 (3.5)	323 (96.4)	335 (39.0)	16 (3.0)	507 (96.9)	523 (60.9)	0.91 (0.40-2.07)	0.82	0	0.98	F
**Neonatal intensive care unit**	4	Beharier etl al. 2021, Blakeway et al. 2022, Rottenstreich M et al. 2021, Theiler et al. 2021	41 (3.8)	1036 (96.1)	1077 (24.1)	80 (2.3)	3310 (97.6)	3390 (75.8)	0.98 (0.66-1.46)	0.08	0	0.76	F
**Small for gestational age**	5	Blakeway et al. 2022, Lipkind et al. 2022, Rottenstreich M et al., 2021, Theiler et al., 2021, Wainstock et al. 2021	869 (8.0)	9957 (91.9)	10826 (21.9)	3018 (7.8)	35491 (92.1)	38509 (78.0)	1.01 (0.93-1.09)	0.82	18	0.30	F
**Stillbirth**	4	Blakeway et al. 2022, Goldshtein et al. 2021, Rottenstreich M et al., 2021, Theiler et al., 2021	6 (0.0)	8509 (99.9)	8515 (43.9)	14 (0.1)	10840 (99.8)	10854 (56.0)	1.13 (0.43-2.97)	0.81	0	0.88	F
**Intrauterine growth restriction**	2	Bleicher et al. 2021, Goldshtein et al. 2021	39 (0.0)	7693 (99.9)	7732 (50.3)	38 (0.0)	7616 (99.9)	7654 (49.7)	1 (0.64-1.57)	1	0	0.32	F
Immunological outcomes													
**COVID-19 infection**	6	*Blakeway et al. 2022, Bleicher et al. 2021, Butt et al. 2021, Dagan et al. 2021, Dawood et al. 2021, Morgan et al. 2022, Theiler et al. 2021*	155 (2.2)	6665 (97.7)	6820 (28.6)	807 (4.7)	16203 (95.2)	17010 (71.3)	0.31 (0.18-0.54)	<0.0001[a]	61	0.02	R

NA: Not applicable; *Mean difference (95% CI); a: Suggestive association (P<0.05); **F: Fixed effect model; R: Random effect model**

Supplementary Table 5. Summary of maternal and umbilical cord blood antibody titers (N=19 studies).

**Study name** (Author)	**Measured antibodies** (antibody/ antibody-antigen/ antigen)	**Type of sample: Time of sampling**	**Seropositive in maternal blood** n positive/N tested (antibody-antigen) Antibody concentration		**Seropositive in cord blood** n positive/N tested (antibody-antigen) Antibody concentration	
			Vaccinated pregnant women	Control group	Vaccinated pregnant women	Control group
Ofer Beharier et al., 2021	IgG and IgM (S1, S2, RBD, Nucleocapsid)	**Maternal and fetal blood**: before delivery<br>**Cord blood:** after delivery	NA	NA	NA	NA
Shanes et al., 2021	IgG-RBD<br>IgM-RBD	**Maternal blood**:<br>at delivery	50/52 (IgG-RBD)<br>30/52 (IgM-RBD)	**Non-vaccinated pregnant:**<br>0/116 (IgG-RBD)<br>0/116 (IgM-RBD)	NA	NA
†Caroline Atyeo et al., 2021	Anti-RBD and Anti-S	**Maternal blood**: 3 to 4 weeks after 1st vaccine dose, at 2nd vaccine dose, 2 to 5.5 weeks after 2nd vaccine dose, and at delivery<br>**Cord blood**: at delivery	NA	NA	NA	NA
Bashi et al., 2021	IgG-RBD<br>IgM-S<br>IgG<br>IgM	**Maternal blood**: during delivery<br>Cord blood: after delivery and before the separation of the placenta	NA	NA	51/58 (IgG)<br>0/58 (IgM)	NA
Bookstein Peretz et al., 2021	IgG-RBD	**Maternal blood**: Two weeks - two months after 2nd vaccine dose	96/96 (IgG-RBD)	**Vaccinated nonpregnant:**<br>96/96 (IgG-RBD)	NA	NA
†Gray et al., 2021	Anti-S1, Anti-S2, Anti-S, Anti-RBD	**Maternal blood**:<br>at 1st vaccine dose,<br>at 2nd vaccine dose<br>2-6 weeks after 2nd vaccine dose, and<br>at delivery<br>**Umbilical cord**:<br>at delivery	NA	NA	10/10 (IgG-RBD)<br>10/10 (IgG-S)	NA
Yang et al., 2021	IgG-S	**Maternal blood**: At delivery	917/917 (IgG-S)<br>**Partially and fully vaccinated**<br>n, median (IQR) =<br>1,007, 5.1 (2.7–7.5) (Pfizer)<br>299, 5.7 (3.8–7.6) (Moderna)<br>33, 3.1 (0.7–5.5) (&J/Janssen)<br>**Fully vaccinated:**<br>n, median (IQR) =<br>917, 5.1 (2.7–7.5) (Pfizer)<br>267, 5.8 (4.0–7.6) (Moderna)<br>31, 3.1 (0.9–5.3) (J&J/Janssen)	NA	NA	NA

Zdanowski et al., 2021	IgG-RBD	**Maternal/Cord blood**: At delivery	16/16 (IgG-RBD)<br>mean (SD) =987.37 U/ml (689.4)	NA	16/16 (IgG-RBD)<br>mean (SD) = 1026.51 U/mL (769.25)	NA
Prahl et al., 2021	IgG-RBD<br>IgM-RBD	**Maternal blood**: pre vaccine, 3-4 weeks after 1st vaccine dose, 4-8 weeks after 2nd vaccine dose, and at delivery<br>**Cord blood**: at delivery	**After 1st vaccine dose:**<br>7/7 (IgG-RBD)<br>mean (SD) = 388.6 RFU (224.8)<br>2/7 (IgM-RBD)<br>mean (SD) = 53.3 RFU (50.2)<br>**After 2nd vaccine dose:**<br>12/12 (IgG-RBD)<br>mean (SD) = 3214 RFU (1383)<br>1/12 (IgM-RBD)<br>mean (SD) = 23.8 RFU (17)<br>**At delivery:**<br>18/19 (IgG-RBD)<br>mean (range) = 3235 RFU (10-7811)	NA	**At delivery:**<br>15/17 (IgG-RBD)<br>mean (range) = 2243 RFU (2-4959]	NA
Shen et al., 2022	IgG-RBD	**Maternal/Cord blood:** On the day of delivery	29/29 (IgG-RBD)	NA	29/29 (IgG-RBD)	NA
Collier et al., 2021	IgG-RBD, IgA	**Maternal blood:**<br>Days since 2nd vaccine dose:<br>Median (IQR) =<br>21 (14-36) (*Vaccinated pregnant*)<br>21 (17-27) (*Vaccinated non-pregnant, non-lactating*)<br>26 (19-31) (*Vaccinated lactating*)<br><br>Days since infection:<br>Median (IQR) =<br>41 (15-140) (Infected pregnant)<br>12 (10-20) (infected non-pregnant)	30/30 (IgG-RBD)<br>median = 27601	**Vaccinated non-pregnant, non-lactating** :<br>53/53 (IgG-RBD)<br>median = 37839<br>**Vaccinated lactating**:<br>14/14 (IgG-RBD) median = 23497<br>**Infected pregnant:** 26/26 (IgG-RBD) median = 1321<br>**Infected non-pregnant**:<br>6/6 (IgG-RBD)<br>median = 771	9/9 (IgG-RBD) median = 19873	**Infected pregnant:** 13/14 (IgG-RBD)<br>median = 635
Rottenstreich, A et al., 2021a	IgG-S1, IgG-S2, IgG-S<br>IgG-RBD, IgM	**Maternal/Cord blood**: After delivery	20/20 (IgG-RBD)<br>median (IQR) = 11150 AU/mL (6154–17575)<br>20/20 (IgG-S)<br>median (IQR) = 319 AU/mL (211–1033)<br>6/20 (IgM)	NA	20/20 (IgG-RBD)<br>median (IQR) = 3494 AU/mL (1817–6163)<br>20/20 (IgG-S) median (IQR) = 193 AU/mL (111–260)<br>0/20 (IgM)	NA
Rottenstreich, A et al 2021b	IgG-S1, IgG-S2, IgG-RBD, IgM	**Maternal/Cord blood:** After delivery	171/171 (IgG-RBD)<br>71/171 (IgG-S)<br>30/171 (IgM)	NA	171/171 (IgG-RBD)<br>171/171 (IgG-S)<br>0/171 (IgG-M)	

Nir et al., 2022	IgG-RBD	**Maternal blood**: on admission.<br>**Cord blood:** immediately postpartum	64/64 (IgG-RBD)<br>median (IQR) = 26.1 (22.0–39.7)	**Recovered**:<br>N, median (IQR): 11, 2.6 (0.9 - 3.5) (IgG-RBD)	63/64 (IgG-RBD)<br>median (IQR) = 20.2 (12.7–29.0)<br>**Neonatal spot sample:** 53/55 (IgG–RBD)<br>median (IQR) = 11.0 (7.2 –12.8)	**Recovered**:<br>N, median (IQR): 11, 3.27 (0.5 - 4.6) (IgG-RBD)
Prabhu et al., 2021	IgG-RBD<br>IgM-RBD	**Maternal/Cord blood:** At delivery	106/122 (IgG-RBD)<br>19/122 (IgM-RBD)	NA	89/122 (IgG-RBD)	NA
Kashani-Ligumsky et al., 2021	IgG-S	**Maternal/Cord blood:** At delivery	**Vaccinated and infected**<br>N, mean (SD) = 30, 157 U/ml (112.8) (IgG-S)	**Non-vaccinated and non-infected**<br>0 positive	29/29 (IgG-S), mean (SD) = 224.7 U/ml (64.3)	**Infected women:** 29/29 (IgG-S)<br>mean (SD) = 83.7 U/ml (91.6)<br>**Non-vaccinated and not infected:** 0/21 (IgG-S)
Kugelman et al., 2021	IgG-RBD	**Maternal/Cord blood:** Within 30 minutes of delivery	129/129 (IgG-RBD)<br>median (range) = 1185.2 AU/mL (146.6 - 32415.1)	NA	114/114 (IgG-RBD)<br>median (range) = 3315.7 AU/ml (350.1 - 17643.5)	NA
Mithal et al., 2021	IgG, IgM (S, RBD)	**Maternal/Cord blood:** At delivery	26/27 (IgG-RBD)<br>15/27 (IgM-RBD)	NA	25/28 (IgG-RBD)<br>0/28 (IgM-RBD)	NA
Matsui et al., 2021	IgG-RBD	At delivery	NA	NA	NA	NA

* Included women received vaccine in pre-pregnancy period, †the two studies had the same population; however, they reported different outcomes, RFU = Relative Fluorescence Units, MFI = the mean fluorescence intensity, and NA = Not Available

Supplementary Figure 1. Forest plot of the odds ratio of non-operative (unassisted) vaginal delivery in vaccinated pregnant women vs. unvaccinated pregnant women using Mantel-Haenszel.

	Vaccinated pregnant		Non-vaccinated pregnant			Odds Ratio
**Study or Subgroup**	**Events**	**Total**	**Events**	**Total**	**Weight**	**M-H, Fixed, 95% CI**
Blakeway et al, 2022	71	133	221	399	39.1%	0.92 [0.62, 1.37]
Kashani-Ligumsky et al, 2021	26	29	18	21	1.6%	1.44 [0.26, 7.98]
Rottenstreich M et al, 2021	11	712	19	1063	11.4%	0.86 [0.41, 1.82]
Theiler et al, 2021	89	140	1238	1862	47.9%	0.88 [0.62, 1.26]
**Total (95% CI)**		**1014**		**3345**	**100.0%**	**0.90 [0.71, 1.16]**
Total events	197		1496			

Heterogeneity: Chi² = 0.34, df = 3 (P = 0.95); I² = 0%
Test for overall effect: Z = 0.80 (P = 0.42)

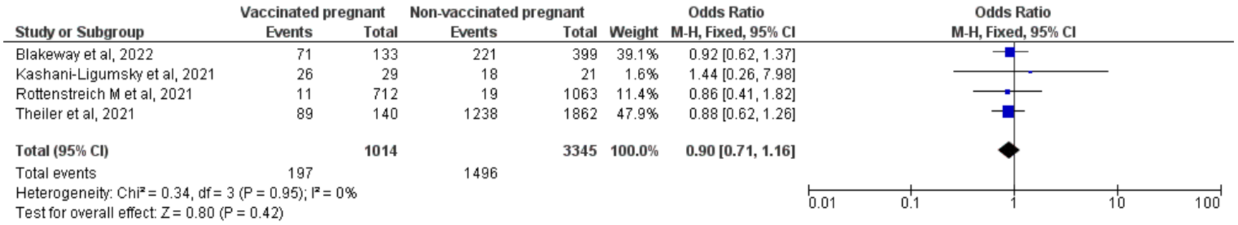

Supplementary Figure 2. Forest plot of the odds ratio of operative vaginal delivery in vaccinated pregnant women vs. unvaccinated pregnant women using Mantel-Haenszel. (A. Main meta-analysis, B. Meta-analysis after the exclusion of the study contributing to heterogeneity).

A

Study or Subgroup	Vaccinated pregnant Events	Vaccinated pregnant Total	Non-vaccinated pregnant Events	Non-vaccinated pregnant Total	Weight	Odds Ratio M-H, Random, 95% CI
Blakeway et al, 2022	21	133	42	399	24.9%	1.59 [0.91, 2.80]
Rottenstreich M et al, 2021	23	712	66	1063	27.0%	0.50 [0.31, 0.82]
Theiler et al, 2021	7	140	69	1862	19.2%	1.37 [0.62, 3.04]
Wainstock et al, 2021	28	913	134	3486	28.9%	0.79 [0.52, 1.20]
**Total (95% CI)**		**1898**		**6810**	**100.0%**	**0.93 [0.55, 1.55]**
Total events	79		311			

Heterogeneity: $Tau^2 = 0.19$; $Chi^2 = 10.75$, df = 3 (P = 0.01); $I^2 = 72\%$
Test for overall effect: Z = 0.29 (P = 0.77)

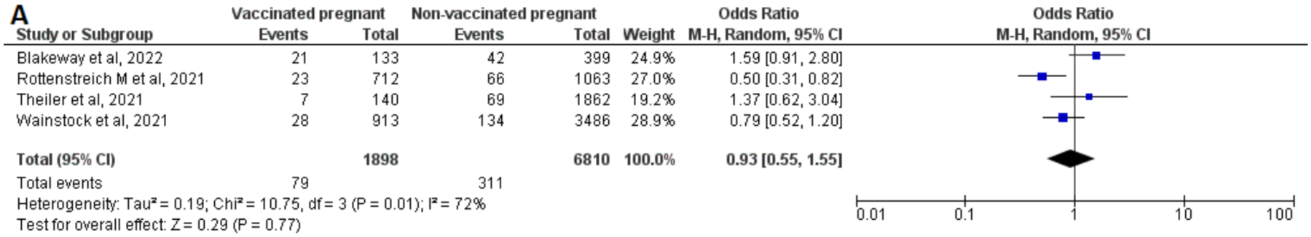

B

Study or Subgroup	Vaccinated pregnant Events	Vaccinated pregnant Total	Non-vaccinated pregnant Events	Non-vaccinated pregnant Total	Weight	Odds Ratio M-H, Random, 95% CI
Blakeway et al, 2022	21	133	42	399	33.8%	1.59 [0.91, 2.80]
Rottenstreich M et al, 2021	23	712	66	1063	0.0%	0.50 [0.31, 0.82]
Theiler et al, 2021	7	140	69	1862	23.3%	1.37 [0.62, 3.04]
Wainstock et al, 2021	28	913	134	3486	42.9%	0.79 [0.52, 1.20]
**Total (95% CI)**		**1186**		**5747**	**100.0%**	**1.14 [0.70, 1.85]**
Total events	56		245			

Heterogeneity: $Tau^2 = 0.10$; $Chi^2 = 4.33$, df = 2 (P = 0.11); $I^2 = 54\%$
Test for overall effect: Z = 0.52 (P = 0.60)

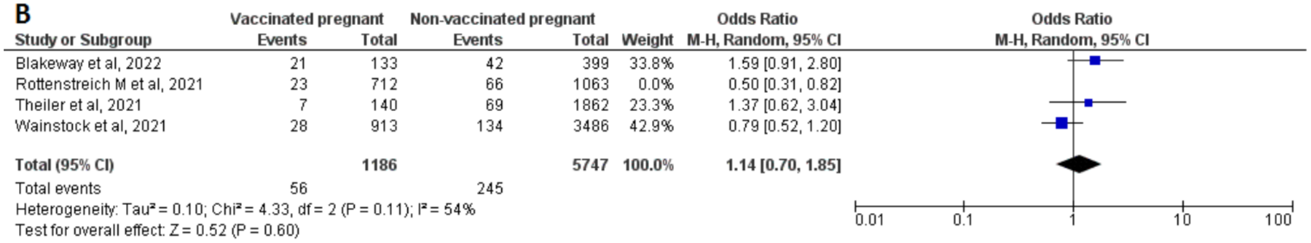

Supplementary Figure 3. Forest plot of the odds ratio of gestational diabetes in vaccinated pregnant women vs. unvaccinated pregnant women using Mantel-Haenszel.

Study or Subgroup	Vaccinated pregnant Events	Vaccinated pregnant Total	Non-vaccinated pregnant Events	Non-vaccinated pregnant Total	Weight	Odds Ratio M-H, Fixed, 95% CI
Blakeway et al, 2022	20	140	146	1188	26.7%	1.19 [0.72, 1.97]
Wainstock et al, 2021	63	913	187	3486	73.3%	1.31 [0.97, 1.76]
**Total (95% CI)**		**1053**		**4674**	**100.0%**	**1.28 [0.99, 1.65]**
Total events	83		333			

Heterogeneity: $Chi^2 = 0.10$, df = 1 (P = 0.75); $I^2 = 0\%$
Test for overall effect: Z = 1.88 (P = 0.06)

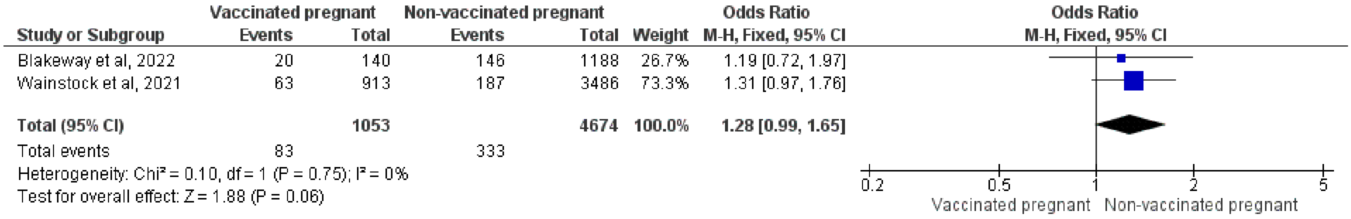

Supplementary Figure 4. Forest plot of the odds ratio of gestational hypertension in vaccinated pregnant women vs. unvaccinated pregnant women using Mantel-Haenszel.

	Vaccinated pregnant		Non-vaccinated pregnant			Odds Ratio
**Study or Subgroup**	**Events**	**Total**	**Events**	**Total**	**Weight**	**M-H, Fixed, 95% CI**
Theiler et al, 2021	19	140	225	1862	29.6%	1.14 [0.69, 1.89]
Wainstock et al, 2021	50	913	165	3486	70.4%	1.17 [0.84, 1.61]
**Total (95% CI)**		**1053**		**5348**	**100.0%**	**1.16 [0.88, 1.52]**
Total events	69		390			

Heterogeneity: Chi² = 0.00, df = 1 (P = 0.95); I² = 0%
Test for overall effect: Z = 1.06 (P = 0.29)

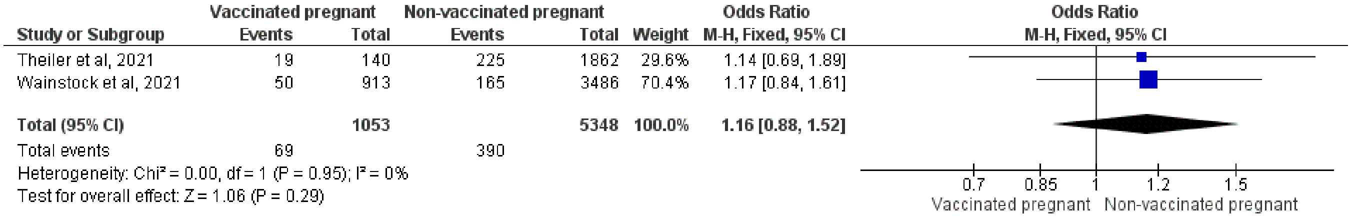

Supplementary Figure 5. Forest plot of the odds ratio of placental abruption in vaccinated pregnant women vs. unvaccinated pregnant women in using Mantel-Haenszel.

Study or Subgroup	Vaccinated pregnant Events	Vaccinated pregnant Total	Non-vaccinated pregnant Events	Non-vaccinated pregnant Total	Weight	Odds Ratio M-H, Fixed, 95% CI
Blakeway et al, 2022	0	133	0	399		Not estimable
Rottenstreich M et al, 2021	8	712	25	1063	81.3%	0.47 [0.21, 1.05]
Wainstock et al, 2021	3	913	11	3486	18.7%	1.04 [0.29, 3.74]
**Total (95% CI)**		**1758**		**4948**	**100.0%**	**0.58 [0.29, 1.14]**
Total events	11		36			

Heterogeneity: Chi² = 1.06, df = 1 (P = 0.30); I² = 6%
Test for overall effect: Z = 1.58 (P = 0.11)

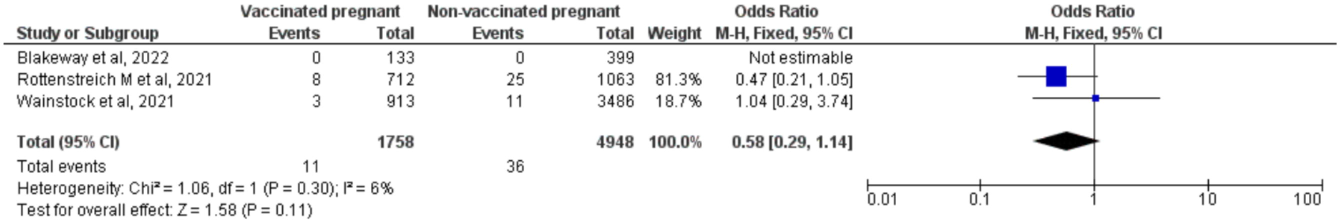

Supplementary Figure 6. Forest plot of the odds ratio of Postpartum Hemorrhage in vaccinated pregnant women vs. unvaccinated pregnant women in using Mantel-Haenszel.

	Vaccinated pregnant		Non-vaccinated pregnant			Odds Ratio
Study or Subgroup	Events	Total	Events	Total	Weight	M-H, Fixed, 95% CI
Blakeway et al, 2022	13	133	36	399	15.0%	1.09 [0.56, 2.13]
Rottenstreich M et al, 2021	52	712	106	1063	72.9%	0.71 [0.50, 1.01]
Theiler et al, 2021	1	140	5	1862	0.6%	2.67 [0.31, 23.03]
Wainstock et al, 2021	10	913	30	3486	11.4%	1.28 [0.62, 2.62]
**Total (95% CI)**		**1898**		**6810**	**100.0%**	**0.85 [0.64, 1.12]**
Total events	76		177			

Heterogeneity: Chi² = 3.88, df = 3 (P = 0.28); I² = 23%
Test for overall effect: Z = 1.17 (P = 0.24)

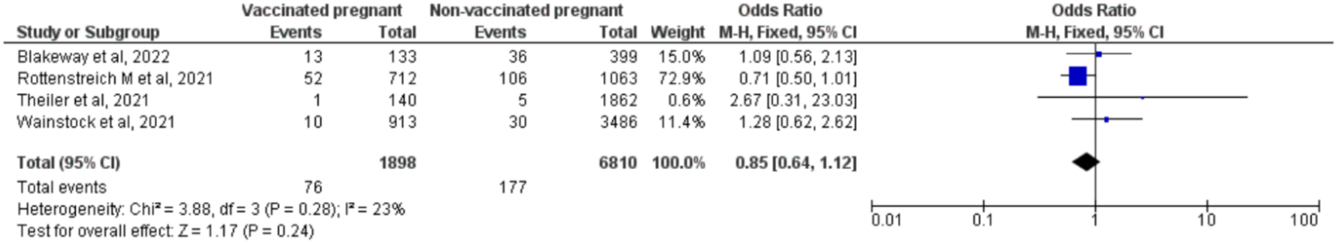

Supplementary Figure 7. Forest plot of the length of hospital stay at the time of delivery in vaccinated pregnant women vs. unvaccinated pregnant women using Inverse Variance. (A. Main meta-analysis, B. Meta-analysis after the exclusion of study contributing to heterogeneity).

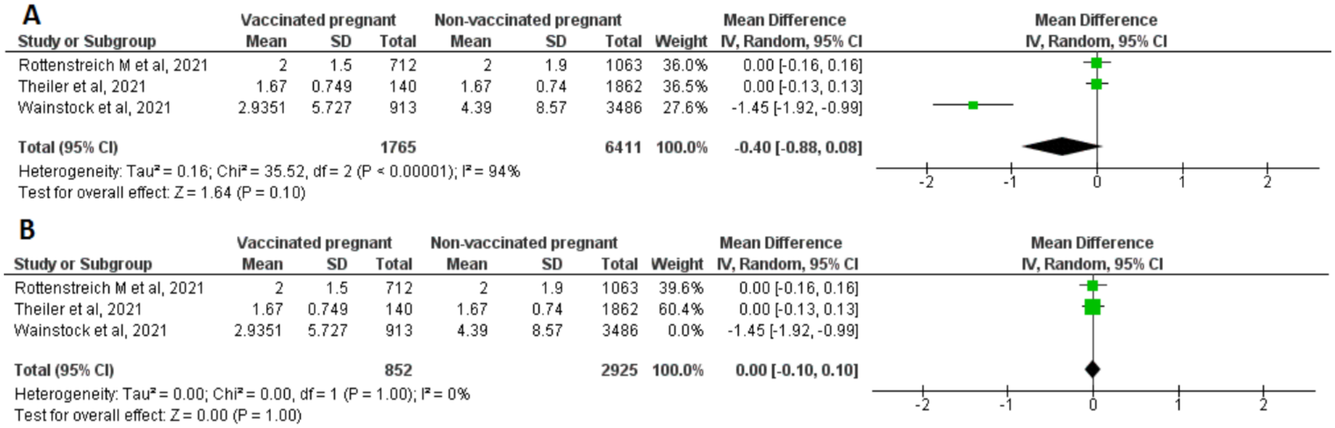

Supplementary Figure 8. Forest plot of the preeclampsia in vaccinated pregnant women vs. unvaccinated pregnant women in using Mantel-Haenszel.

	Vaccinated pregnant		Non-vaccinated pregnant			Odds Ratio
**Study or Subgroup**	**Events**	**Total**	**Events**	**Total**	**Weight**	**M-H, Fixed, 95% CI**
Goldshtein et al, 2021	20	7530	21	7530	86.8%	0.95 [0.52, 1.76]
Theiler et al, 2021	1	140	23	1862	13.2%	0.58 [0.08, 4.29]
**Total (95% CI)**		**7670**		**9392**	**100.0%**	**0.90 [0.50, 1.61]**
Total events	21		44			

Heterogeneity: Chi² = 0.22, df = 1 (P = 0.64); I² = 0%

Test for overall effect: Z = 0.35 (P = 0.73)

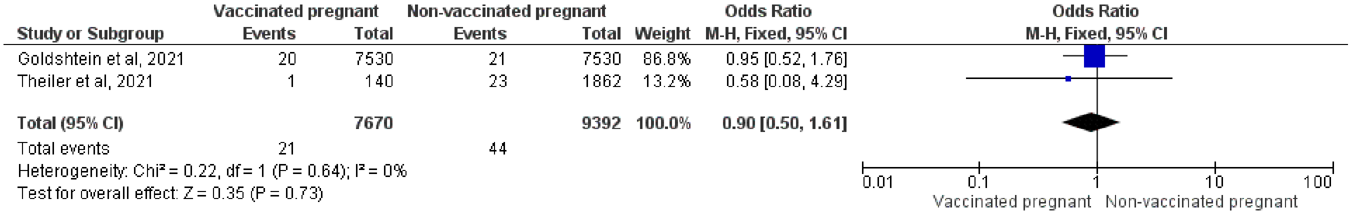

Supplementary Figure 9. Forest plot of five minutes Apgar score less than 7 in vaccinated pregnant women vs. unvaccinated pregnant women in using Mantel-Haenszel.

	Vaccinated pregnant		Non-vaccinated pregnant			Odds Ratio
Study or Subgroup	Events	Total	Events	Total	Weight	M-H, Fixed, 95% CI
Rottenstreich M et al, 2021	21	712	27	1063	54.4%	1.17 [0.65, 2.08]
Theiler et al, 2021	3	140	38	1862	13.5%	1.05 [0.32, 3.45]
Wainstock et al, 2021	2	913	30	3486	32.2%	0.25 [0.06, 1.06]
**Total (95% CI)**		**1765**		**6411**	**100.0%**	**0.86 [0.54, 1.37]**
Total events	26		95			

Heterogeneity: Chi² = 3.99, df = 2 (P = 0.14); I² = 50%
Test for overall effect: Z = 0.64 (P = 0.52)

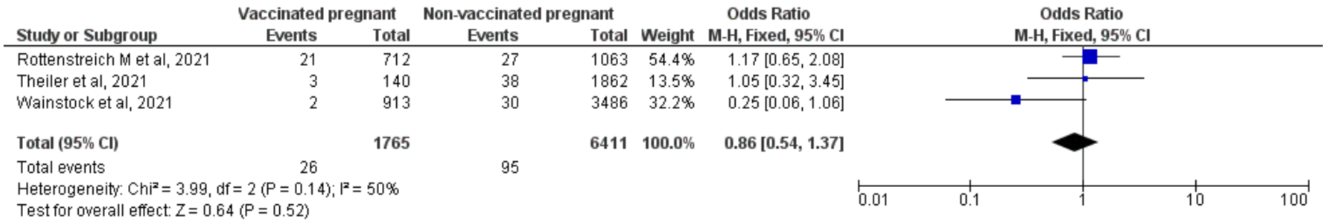

Supplementary Figure 10. Forest plot of first-trimester miscarriage in vaccinated pregnant women vs. unvaccinated pregnant women in using Mantel-Haenszel.

	Vaccinated pregnant		Non-vaccinated pregnant			Odds Ratio
Study or Subgroup	Events	Total	Events	Total	Weight	M-H, Fixed, 95% CI
Goldshtein et al, 2021	128	7530	118	7530	24.4%	1.09 [0.84, 1.40]
Magnus et al, 2021	231	1003	4290	17474	75.6%	0.92 [0.79, 1.07]
**Total (95% CI)**		**8533**		**25004**	**100.0%**	**0.96 [0.84, 1.09]**
Total events	359		4408			

Heterogeneity: Chi² = 1.23, df = 1 (P = 0.27); I² = 19%

Test for overall effect: Z = 0.62 (P = 0.54)

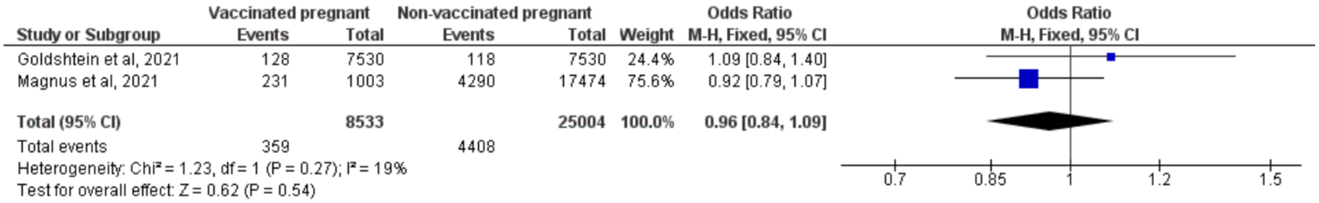

Supplementary Figure 11. Forest plot of fetal abnormalities in vaccinated pregnant women vs. unvaccinated pregnant women in using Mantel-Haenszel.

	Vaccinated pregnant		Non-vaccinated pregnant			Odds Ratio
**Study or Subgroup**	**Events**	**Total**	**Events**	**Total**	**Weight**	**M-H, Fixed, 95% CI**
Blakeway et al, 2022	3	133	10	399	40.8%	0.90 [0.24, 3.31]
Bleicher et al, 2021	9	202	6	124	59.2%	0.92 [0.32, 2.64]
**Total (95% CI)**		**335**		**523**	**100.0%**	**0.91 [0.40, 2.07]**
Total events	12		16			

Heterogeneity: $Chi^2 = 0.00$, $df = 1$ ($P = 0.98$); $I^2 = 0\%$
Test for overall effect: $Z = 0.23$ ($P = 0.82$)

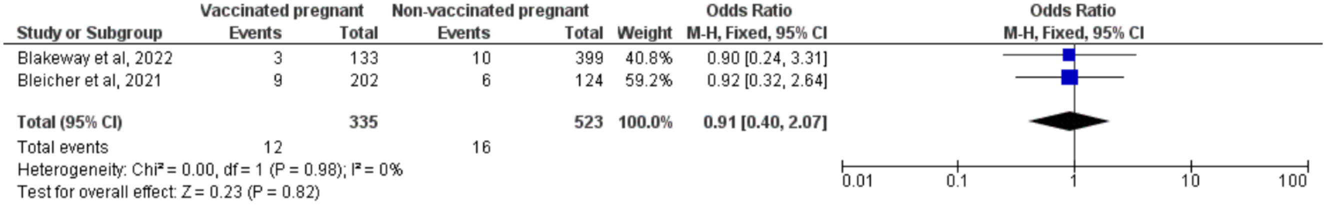

Supplementary Figure 12. Forest plot of neonatal intensive care unit admission in vaccinated pregnant women vs. unvaccinated pregnant women using Mantel-Haenszel.

Study or Subgroup	Vaccinated pregnant Events	Vaccinated pregnant Total	Non-vaccinated pregnant Events	Non-vaccinated pregnant Total	Weight	Odds Ratio M-H, Fixed, 95% CI
Beharier et al, 2021	4	92	1	66	2.3%	2.95 [0.32, 27.06]
Blakeway et al, 2022	7	133	20	399	19.3%	1.05 [0.43, 2.55]
Rottenstreich M et al, 2021	29	712	48	1063	75.3%	0.90 [0.56, 1.44]
Theiler et al, 2021	1	140	11	1862	3.1%	1.21 [0.16, 9.44]
**Total (95% CI)**		**1077**		**3390**	**100.0%**	**0.98 [0.66, 1.46]**
Total events	41		80			

Heterogeneity: Chi² = 1.15, df = 3 (P = 0.76); I² = 0%
Test for overall effect: Z = 0.08 (P = 0.94)

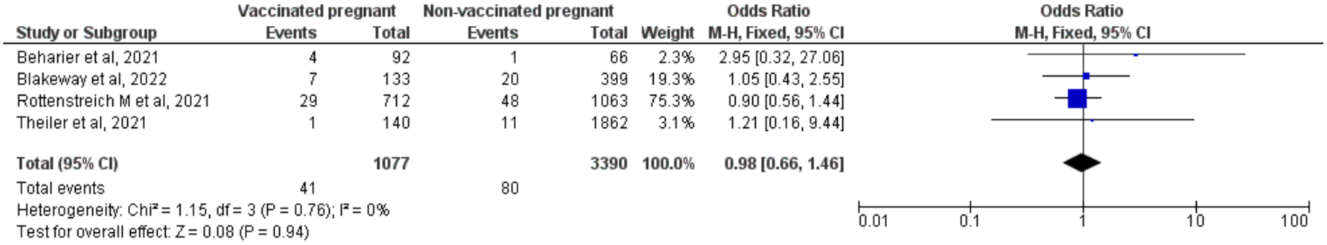

Supplementary Figure 13. Forest plot of small for gestational age in vaccinated pregnant women vs. unvaccinated pregnant women using Mantel-Haenszel.

	Vaccinated pregnant		Non-vaccinated pregnant			Odds Ratio
**Study or Subgroup**	**Events**	**Total**	**Events**	**Total**	**Weight**	**M-H, Fixed, 95% CI**
Blakeway et al, 2022	16	133	48	399	1.7%	1.00 [0.55, 1.83]
Lipkind et al, 2022	732	8928	2599	31699	86.7%	1.00 [0.92, 1.09]
Rottenstreich M et al, 2021	81	712	98	1063	5.8%	1.26 [0.93, 1.73]
Theiler et al, 2021	14	140	142	1862	1.5%	1.35 [0.75, 2.40]
Wainstock et al, 2021	26	913	131	3486	4.4%	0.75 [0.49, 1.15]
**Total (95% CI)**		**10826**		**38509**	**100.0%**	**1.01 [0.93, 1.09]**
Total events	869		3018			

Heterogeneity: Chi² = 4.85, df = 4 (P = 0.30); I² = 18%
Test for overall effect: Z = 0.23 (P = 0.82)

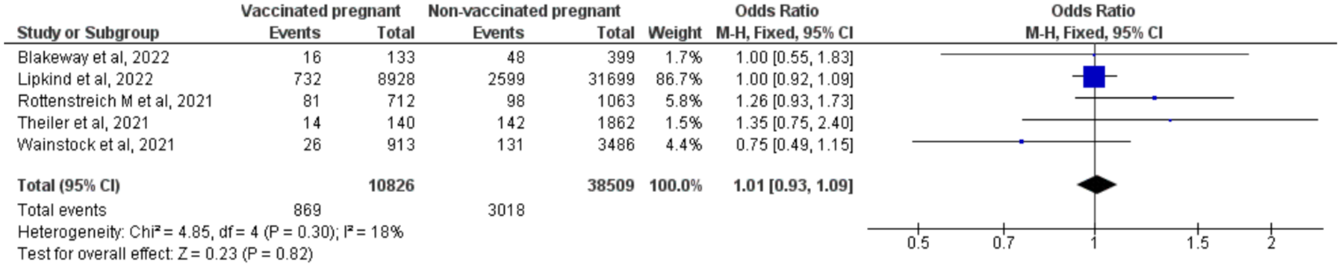

Supplementary Figure 14. Forest plot of intrauterine growth restriction in vaccinated pregnant women vs. unvaccinated pregnant women using Mantel-Haenszel.

	Vaccinated pregnant		Non-vaccinated pregnant			Odds Ratio
**Study or Subgroup**	**Events**	**Total**	**Events**	**Total**	**Weight**	**M-H, Fixed, 95% CI**
Bleicher et al, 2021	3	202	0	124	1.6%	4.37 [0.22, 85.29]
Goldshtein et al, 2021	36	7530	38	7530	98.4%	0.95 [0.60, 1.50]
**Total (95% CI)**		**7732**		**7654**	**100.0%**	**1.00 [0.64, 1.57]**
Total events	39		38			

Heterogeneity: Chi² = 1.00, df = 1 (P = 0.32); I² = 0%

Test for overall effect: Z = 0.01 (P = 1.00)

Odds Ratio
M-H, Fixed, 95% CI

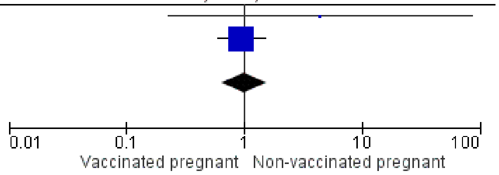

Supplementary Figure 15. Forest plot of stillbirth in vaccinated pregnant women vs. unvaccinated pregnant women using Mantel-Haenszel.

	Vaccinated pregnant		Non-vaccinated pregnant			Odds Ratio
**Study or Subgroup**	**Events**	**Total**	**Events**	**Total**	**Weight**	**M-H, Fixed, 95% CI**
Blakeway et al, 2022	0	133	1	399	9.8%	1.00 [0.04, 24.57]
Goldshtein et al, 2021	1	7530	2	7530	26.2%	0.50 [0.05, 5.51]
Rottenstreich M et al, 2021	5	712	5	1063	52.1%	1.50 [0.43, 5.19]
Theiler et al, 2021	0	140	6	1862	11.9%	1.02 [0.06, 18.13]
**Total (95% CI)**		**8515**		**10854**	**100.0%**	**1.13 [0.43, 2.97]**
Total events	6		14			

Heterogeneity: Chi² = 0.65, df = 3 (P = 0.88); I² = 0%

Test for overall effect: Z = 0.25 (P = 0.81)

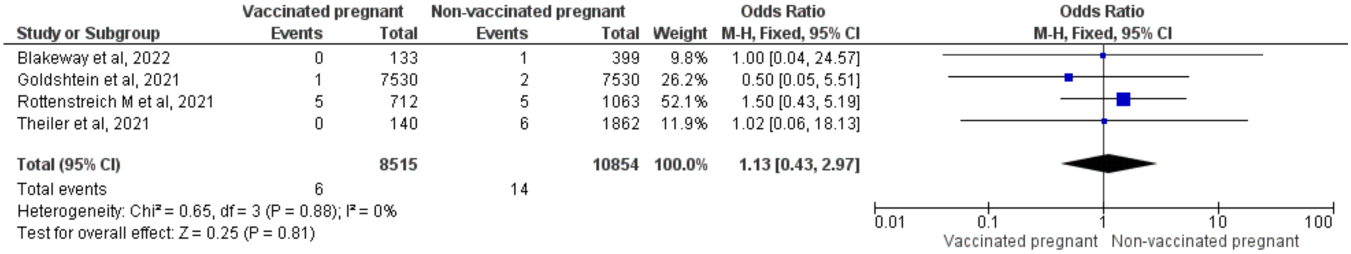

Supplement: Supplementary file 1 — Supplemental Material [file 41541_2023_698_MOESM1_ESM.pdf]
